# Habitat specificity modulates the bacterial biogeographic patterns in the Southern Ocean

**DOI:** 10.1093/femsec/fiae134

**Published:** 2024-10-03

**Authors:** Mélanie Delleuze, Guillaume Schwob, Julieta Orlando, Karin Gerard, Thomas Saucède, Paul Brickle, Elie Poulin, Léa Cabrol

**Affiliations:** Laboratorio de Ecología Molecular, Departamento de Ciencias Ecológicas, Facultad de Ciencias, Universidad de Chile, Santiago 7800003, Chile; Marine Biology Lab, CP160/15, Université Libre de Bruxelles (ULB), Brussels 1050, Belgium; Millennium Institute Biodiversity of Antarctic and Subantarctic Ecosystems (BASE), Santiago 7800003, Chile; Millennium Institute Biodiversity of Antarctic and Subantarctic Ecosystems (BASE), Santiago 7800003, Chile; Millennium Institute Biodiversity of Antarctic and Subantarctic Ecosystems (BASE), Santiago 7800003, Chile; Laboratorio de Ecología Microbiana, Departamento de Ciencias Ecológicas, Facultad de Ciencias, Universidad de Chile, Santiago 7800003, Chile; Millennium Institute Biodiversity of Antarctic and Subantarctic Ecosystems (BASE), Santiago 7800003, Chile; Laboratorio de Ecosistemas Marinos Antárticos y Subantárticos, Universidad de Magallanes, Punta Arenas 6210427, Chile; Cape Horn Investigation Center, Puerto Williams 6350054, Chile; Biogéosciences, UMR CNRS 6282, Université de Bourgogne, 21000 Dijon, France; South Atlantic Environmental Research Institute, Port Stanley FIQQ 1ZZ, Falkland Islands; School of Biological Sciences (Zoology), University of Aberdeen, Aberdeen AB24 3FX, Scotland, United Kingdom; Laboratorio de Ecología Molecular, Departamento de Ciencias Ecológicas, Facultad de Ciencias, Universidad de Chile, Santiago 7800003, Chile; Millennium Institute Biodiversity of Antarctic and Subantarctic Ecosystems (BASE), Santiago 7800003, Chile; Millennium Institute Biodiversity of Antarctic and Subantarctic Ecosystems (BASE), Santiago 7800003, Chile; Aix-Marseille University, Univ Toulon, CNRS, IRD, Mediterranean Institute of Oceanography (M.I.O.) UM 110, 13009 Marseille, France

**Keywords:** microbial biogeography, habitat selectivity, ecological processes, host-associated microbiota, irregular sea urchins, Antarctic

## Abstract

Conceptual biogeographic frameworks have proposed that the relative contribution of environmental and geographical factors on microbial distribution depends on several characteristics of the habitat (e.g. environmental heterogeneity, species diversity, and proportion of specialist/generalist taxa), all of them defining the degree of habitat specificity, but few experimental demonstrations exist. Here, we aimed to determine the effect of habitat specificity on bacterial biogeographic patterns and assembly processes in benthic coastal ecosystems of the Southern Ocean (Patagonia, Falkland/Malvinas, Kerguelen, South Georgia, and King George Islands), using 16S rRNA gene metabarcoding. The gradient of habitat specificity resulted from a ‘natural experimental design’ provided by the *Abatus* sea urchin model, from the sediment (least specific habitat) to the intestinal tissue (most specific habitat). The phylogenetic composition of the bacterial communities showed a clear differentiation by site, driven by a similar contribution of geographic and environmental distances. However, the strength of this biogeographic pattern decreased with increasing habitat specificity: sediment communities showed stronger geographic and environmental divergence compared to gut tissue. The proportion of stochastic and deterministic processes contributing to bacterial assembly varied according to the geographic scale and the habitat specificity level. For instance, an increased contribution of dispersal limitation was observed in gut tissue habitat. Our results underscore the importance of considering different habitats with contrasting levels of specificity to better understand bacterial biogeography and assembly processes over oceanographic scales.

## Teaser

The strength of the microbial biogeographic patterns decreased along the increasing gradient of habitat specificity (from sediment to gut tissue) provided by a benthic sea urchin in the Southern Ocean.

## Introduction

Understanding how bacterial communities are distributed across space and time, and which mechanisms are behind their distribution, is crucial for gaining deeper insights into marine ecosystem biogeography. For a long time, it has been suggested that microorganisms were broadly distributed, and that environmental selection (also known as species sorting) was the only process driving bacterial assemblages (Baas-Becking 1934). However, this hypothesis has been challenged in the last decades with solid evidence of bacterial biogeographic patterns driven by both local environmental conditions and historical dispersal-related factors (Martiny et al. 2006, Langenheder and Lindström 2019). Four processes (selection, drift, dispersal, and mutation) have been proposed to create and maintain bacterial biogeographic patterns at both ecological and evolutionary levels (Vellend 2010, Hanson et al. 2012, Nemergut et al. 2013). In addition to the description of the mechanisms involved in the origin and maintenance of bacterial biodiversity patterns, it is important to determine the conditions that cause a particular mechanism to dominate the assembly processes of bacterial communities. In their conceptual overview, Langenheder and Lindström (2019) established how different factors such as the environmental heterogeneity, the dispersal capacity, the spatial scale, and the considered taxonomic and/or functional groups, could influence the relative contribution of different community assembly processes.

The degree of habitat specificity is also likely an important factor modulating bacterial biogeography, especially in determining the relative contribution of environmental and geographic distances. Specific habitats are defined as habitats with low environmental heterogeneity (i.e. with homogeneous abiotic properties) and are characterized by a low species richness (Wagner and Edwards 2001, Schwob et al. 2020, Liu et al. 2022) and a high proportion of specialist taxa (Hattermann et al. 2019, Liu et al. 2022). Moreover, specific habitats can be considered as island-like habitats, constraining the connectivity and dispersal capacity for bacteria (Loudon et al. 2016, Itescu 2019). All the abovementioned characteristics of habitat specificity might influence the bacterial biogeographic patterns. For instance, habitat specialists taxa (characteristic of homogeneous habitats) are less influenced by species sorting than habitat generalists (Székely and Langenheder 2014). Stronger geographic effects have been reported in habitats, where microorganisms are less prone to dispersion compared to more interconnected habitats (Seppey et al. 2023). Stochastic processes (such as drift and dispersal limitation) are more important when alpha diversity is low and/or in host-associated environments (Chase and Myers 2011, Lankau et al. 2012, Nemergut et al. 2013). It is also well recognized that a low habitat heterogeneity limits the number and diversity of available niches for colonization, generally leading to a lower effect of species selection processes on bacterial assemblage in comparison to stochastic processes (Ramette and Tiedje 2007, Östman et al. 2010, Lindström and Langenheder 2012, Langenheder and Lindström 2019). In addition, the capacity of an organism to successfully colonize and establish in a habitat is related to its niche breadth. The niche breadth represents how generalized or specialized an organism is regarding its habitat requirements.

Assessing the influence of habitat specificity on microbial community assembly could be achieved in laboratory experiments by artificially creating habitats with varying degrees of heterogeneity, species richness (e.g. through sterilization), and connectivity. Laboratory experiments enable to isolate the effect of a single factor while controlling for all others. However, such experiments often include environmental biases and do not realistically reflect the complexity and variability of natural ecosystems (Diamond 1986). In contrast, natural experiments offer a more realistic approach, are not constrained by temporal and spatial scales, and provide more generalizable insights into natural ecological processes (Diamond 1986). In this context, the habitats offered by hosts to their associated microorganisms represent an attractive natural experimental design (Diamond 1986) to compare different levels of habitat specificity across different biogeographic provinces. Indeed, when comparing different locations across a large spatial scale, the habitats associated with phylogenetically and ecologically closely related hosts tend to exbibit more homogenous environmental conditions than the surrounding open environments, due to the selective pressures imposed by the hosts. Thus, the importance of species sorting across different sites might be less important in host-associated habitats (due to their higher homogeneity) compared to nonhost associated habitats (Langenheder and Lindström 2019). The host filtering conditions tend to select more phylogenetically related taxa, acquired either from the environment or through vertical transmission from parent to offspring (Carrier et al. 2024). Even if vertical transmission is an important factor in structuring some host-associated microbiota (Sharp et al. 2007, Baldassarre et al. 2021, Carrier et al. 2021), gut microbiota are dynamics and expected to change throughout the individual’s life, in close relation with environmental factors and diet (Carrier et al. 2021, Masasa et al. 2021, Renelies-Hamilton et al. 2021, Kang et al. 2022), especially in deposit-feeding hosts that are continuously flushed by their feed matrix (e.g. soil or sediment, in a plug-flow mode) and thus exposed to environmental microbial communities. Moreover, host-resident bacteria would less easily disperse and establish across large spatial scales compared to free-living bacteria, because they are constrained by the biotic and abiotic conditions imposed by the host habitat. For instance, strictly anaerobic microbes show high specificity for host environments (Mazel et al. 2024). This should lead to a greater effect of spatial distance on community assembly associated to hosts. Recently, in different Australian estuaries, Suzzi et al. (2023) compared host-associated and free-living microbiota. They showed that bacterial communities from fish hindguts (here considered as a presumably more homogeneous habitat) were less constrained by environmental selection and geographic distance than bacterial communities from seawater and sediments (considered here as more heterogeneous habitats) (Suzzi et al. 2023). However, the influence of habitat specificity level on ecological assembly processes (selection, drift and dispersal) remains to be investigated.

The irregular deposit-feeding sea urchin genus *Abatus* (Troschel 1851) represents a suitable host model for bacterial biogeographic studies, as it is distributed across different biogeographic provinces of the Southern Ocean. While some *Abatus* species are separated by strong natural marine biogeographic barriers (e.g. the Antarctic Polar Front; APF), others are distributed in distant regions connected by the Antarctic Circumpolar Current (ACC). These sea urchins are infaunal organisms living at shallow depths buried in medium to very fine muddy sand sediments (David et al. 2005), feeding on the organic matter from the ingested sediment, and they are considered important bioturbators of the ocean floor (Lohrer et al. 2005). Sea urchins from the genus *Abatus* are generally easily accessible for sampling and are found in high-density patchy populations (Poulin and Féral 1995, Gil et al. 2009). Thanks to their feeding behaviour, *Abatus* sea urchins naturally provide a gradient of habitat specificity, from most to least specific: (i) the *Abatus* gut tissue habitat, (ii) the ingested sediment in gut content, and (iii) the external sediment habitat, which constitutes the sea urchins feeding source. Indeed, the microbial communities of these three habitats have been previously shown to be drastically different, with lower diversity and more specialist taxa in the gut tissue, which is characteristic of specific habitats (Schwob et al. 2020, Ziegler et al. 2020). Thus, the *Abatus* sea urchin provides a unique ‘natural experiment’ model to test how different levels of habitat specificity can modulate the bacterial biogeographic patterns across a large oceanographic scale.

In this study, we aim to determine the influence of habitat specificity on the bacterial biogeographic patterns and assembly processes in benthic ecosystems from five localities of the Southern Ocean, distributed across three biogeographic regions according to the definition of Koubbi et al. (2014): Magellanic [(i)Patagonia and (ii) Falkland/Malvinas Islands], Subantarctic [(iii) Kerguelen Islands], and Antarctic [(iv) South Shetland Islands and (v) South Georgia] provinces. We hypothesize that (i) within each habitat (i.e. sediment, gut content, and gut tissue), the bacterial communities structure will differ across regions due to the interplay between geographical and environmental factors, and (ii) the strength of these biogeographical patterns and their underlying ecological assembly processes will be modulated by the level of habitat specificity, due to the distinct degree of dispersal capacity offered by each habitat. In particular, as they are directly exposed to the environment, the sediment bacteria are expected to be more prone to dispersal and environmental filtering than sea urchin gut tissue bacteria. This suggests that microorganisms associated to the sediment would show stronger biogeographic patterns than the ones associated to the gut tissue, while those associated to the gut contents would display an intermediate pattern.

## Materials and methods

### Natural experimental design

**Figure 1.**
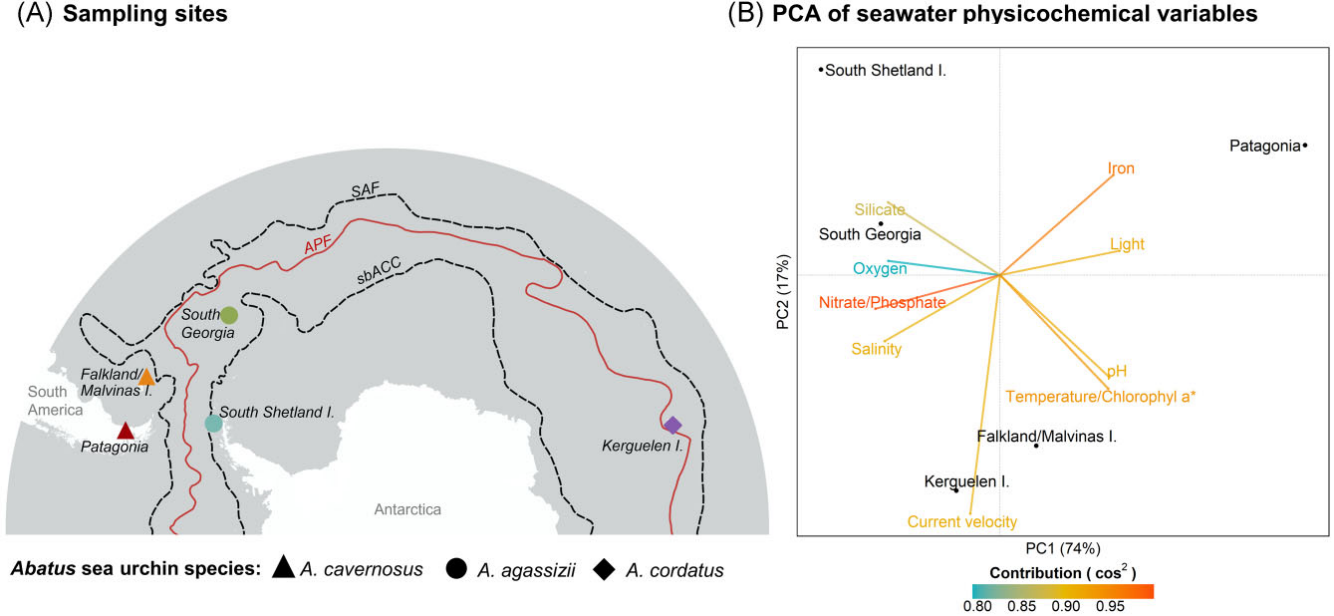
Sampling sites localities and environmental characterization across the Southern Ocean. (A) Map showing the sampling sites: Possession Bay in Atlantic Patagonia, Canache Bay in Falkland/Malvinas Islands, Fildes Bay in King George Island, South Shetland Islands, Stromness Bay in South Georgia, and Port-aux-Français in Kerguelen Islands. The different *Abatus* species are indicated by the symbol shapes. Some localities are connected by the ACC characterized here by its north and south limits (black dashed lines): the Subantarctic Front (SAF) and the southern boundary of the ACC (sbACC). Other localities are separated by the APF (continuous red line). The positions of the fronts were extracted from Orsi et al. (1995). (B) Principal component analysis (PCA) of the seawater physicochemical variables from each sampling site. The arrows represent the environmental values and are coloured according to their contribution to the PCA based on the cos^2^. Variables that were highly correlated (Pearson correlation coefficient >0.95) were represented by the same arrow. Here, ‘Temperature/Chlorophyl a*’ represents four correlated variables (temperature, chlorophyll a concentration, mean net primary productivity of carbon, and carbon phytoplankton biomass).

The natural experimental design of this study consists of three habitats naturally provided by *Abatus* sea urchin: (i) gut tissue habitat (i.e. the whole intestinal membrane except the caecum), (ii) the gut content habitat (i.e. the ingested sediment within the digestive tract) (Schwob et al. 2020), and (iii) the external sediment habitat, which constitutes the sea urchin feeding source. *Abatus* specimens of three different species (*A. agassizii, A. cavernosus*, and *A. cordatus*) were collected from five localities of the Southern Ocean. These species are the most ecologically and phylogenetically closely related among the eleven existing *Abatus* species (David et al. 2005, Díaz et al. 2012). Thanks to their similar ecology and sediment-feeding behaviour, the different species provide the same natural gradient of habitats in different biogeographic provinces of the Southern Ocean. The five studied localities span over three biogeographic provinces according to the definition of Koubbi et al. (2014): the Magellanic province (Patagonia and Falkland/Malvinas Islands, where *A. cavernosus* lives), Subantarctic islands province of the southern Indian Ocean (Kerguelen Island, where *A. cordatus* occurs), and the Antarctic province (South Shetland Islands and South Georgia, where *A. agassizii* is found) (Table 1; Fig. 1A). A single *Abatus* species is found in each site. Two sites accommodate the same *Abatus* species (Table 1).

**Table 1.** Study design. Sampling sites of adult sea urchin genus *Abatus* and of the surrounding sediment across the Southern Ocean. (*N*: number of samples). The benthic biogeographic regions according to Koubbi et al. (2014) are indicated for each site and are consistent with the host species found in each of them.

Designation	Site	Province	Biogeographic regions	GPS coordinates	Date	*Abatus* species	Sampling method	Depth (m)	Habitat	*N*
Patagonia	Possession Bay, Magellan Strait	SubAntarctic	Magellan	52°19′52.97° S 69°29′10.50° W	19 July	*A. cavernosus*	Manual collection at low tide	0.1	Sediment	6
									Gut content	37
									Gut tissue	15
Falkland/ Malvinas I.	Canache Bay	SubAntarctic	Magellan	51°41´45.84° S 57°47´8.73° W	22 September	*A. cavernosus*	Manual collection at low tide	0.1	Sediment	5
									Gut content	15
									Gut tissue	12
South Georgia	Stromness Bay	Antarctic	Maritime Antarctic	54°9′43.2° S 36°39′50.4° W	21 November	*A. agassizii*	Scuba diving	16	Sediment	6
									Gut content	7
									Gut tissue	9
South Shetland I.	Fildes Bay, King George Island, South Shetland Islands	Antarctic	Maritime Antarctic	62°13′7.644° S 58°57′28.295° W	19 January	*A. agassizii*	Scuba diving	2	Sediment	6
									Gut content	36
									Gut tissue	20
Kerguelen I.	Port-aux-Français, Morbihan Bay	SubAntarctic	SubAntarctic Islands South Indian Ocean	49°21′13.32° S 70°13′8.759° E	17 November	*A. cordatus*	Scuba diving	3	Sediment	5
									Gut content	15
									Gut tissue	6
									**Total**	200

The five studied localities (Table 1, Fig. 1) exhibit varying environmental conditions and are positioned in different biogeographic provinces of the Southern Ocean as detailed in De Broyer et al. (2014). In the maritime Antarctic, King George Island, located north of the Shetland Islands, experiences cold temperatures ranging from below 10°C to 0–2°C, with notable seasonal ice changes affecting ecosystem dynamics (Ducklow et al. 2013). South Georgia Island, situated north of the Scotia Arc and south of the APF, is part of the Antarctic biogeographic province (Spalding et al. 2007, Koubbi et al. 2014), its climate is polar, and the weather is highly variable and harsh, with temperature ranging from −10°C to 8°C. In Atlantic Patagonia, the Strait of Magellan is characterized by strong tidal currents, winds, and low-salinity waters (Medeiros and Kjerfve 1988, Brun et al. 2020). The Falkland Islands have a cool, temperate oceanic climate, characterized by mild summers and cold, windy winters, and are part of the Magellan biogeographic province (Spalding et al. 2007, Koubbi et al. 2014). Lastly, the Kerguelen Archipelago, particularly Port-aux-Français, features a cold oceanic climate typical of subAntarctic islands.

### Samples collection and treatment

Sampling was conducted across the five localities of the Southern Ocean between November 2017 and September 2022, during the austral summer (Table 1). Sampled individuals were immediately stored in a reservoir containing *in situ* seawater and sediment at 4°C until dissection. Superficial coastal sediment from the immediate surroundings of the sea urchins was also collected and stored at 4°C until laboratory treatment. In the laboratory, between 2 and 12 h after sampling, the sea urchins (*n*_min =_ 6, *n*_max_ = 20; Table 1) were dissected under sterile conditions to collect separately the gut content and the gut tissue The gut tissue was gently rinsed in sterile ultrapure DNAse-free water (Winkler, Chile) to remove all the ingested sediment. The external sediment was homogenized and aliquoted in five to six replicates. All the samples were stored separately in 2 ml cryotubes at −20°C until DNA extraction.

### Environmental and geographic distance data

For each sampling site, 13 environmental parameters obtained through modelling were extracted from the Bio-ORACLE 2.2 dataset (Assis et al. 2018), including nutrient concentrations (iron, phosphate, nitrate, and silicate), productivity measures (chlorophyll a concentration, mean net primary productivity of carbon, and carbon phytoplankton biomass), and physical–chemical parameters of the seawater (temperature, salinity, pH, current velocity, and light penetration). The mean environmental measures were taken at the mean depth for each location and were centre-reduced ((*x*_i_ − *x̅*) ⁄ σ_*x*_). For highly correlated variables (Pearson correlation coefficient >0.95), only one representative was kept for further analysis.

The environmental distance matrix was calculated based on Euclidean distance from the normalized uncorrelated environmental variables (*dist* function, *stats* package 4.3.2) and used to run a principal component analysis (PCA; *prcomp* function, *stats* package). As a high percentage of variance were explained by the first two components (91%; Fig. 1B), the sample scores on PC1 and PC2 axis were converted in a new PCA-based environmental distance matrix based on Euclidean distance.

The geographic distance matrix was calculated from the Global Positioning System (GPS) coordinates of each site determined on an ellipsoid map (function *distGeo*, package *geosphere* version 1.5–18). The spatial structure was represented by the three principal coordinates of a neighbourhood matrix (Borcard and Legendre 2002) with a truncation distance threshold equal to the maximum geographical distance (PCNM1-3, function *pcnm, vegan* package; Oksanen et al. 2007). The principal coordinates (PCNM variables) derived from these eigenvalues were used as explanatory geographic variables for further analysis.

### DNA extraction, 16S rRNA gene amplification, and sequencing

Defrosted samples of external sediment, gut content, and gut tissue were homogenized, and DNA was extracted from a maximum of 350 mg of sample using the DNeasy® PowerSoil® Pro Kit (Qiagen, Hilden, Germany) following the manufacturer’s indications, except for gut tissue samples where the cell lysis was carried out in the FastPrep-24® homogenizer (3 times 15 s at 4 m s^−1^) (MP Biomedicals, USA). The extracted genomic DNA was quantified using the Qubit® 3.0. Fluorometer (ThermoFisher Scientific, Lithuania).

The V4–V5 hypervariable region of the 16S rRNA gene was amplified with the primers 515F (5′-GTGYCAGCMGCCGCGGTA-3′) and 928R (5′- CCCCGYCAATTCMTTTRAGT-3′) (Wang and Qian 2009). The Polymerase Chain Reaction (PCR) mix contained 2–5 μl of undiluted template DNA (10–200 ng µl^−1^), 0.3 µM of each primer, 25 μl of Phusion Hot Start II High-Fidelity PCR MasterMix (ThermoFisher Scientific), and nuclease-free water (q.s. 50 μl) (ThermoFisher Scientific). The amplification conditions were an initial denaturation at 98°C for 3 min, 35 two-step cycles of 10 s at 98°C and 15 s at 72°C for annealing and elongation, and a final extension step of 5 min at 72°C, as in Schwob et al. (2020). The quantity and quality of the PCR products were checked on agarose electrophoresis gels.

The amplicons were sequenced at the DNA sequencing facility of the University of Wisconsin-Madison Biotechnology Centre (UWBC, USA) using the paired-end sequencing technology (2 × 300 bp) with the V2 chemistry on Illumina MiSeq.

The fastq files from Falkland/Malvinas Islands and South Georgia (generated in this study) are available at the National Center for Biotechnology Information (NCBI) under the project accession number PRJNA1065352. The fastq files from Antarctica, Patagonia, and Kerguelen sites have been extracted from previously published works (Schwob et al. 2020, 2021), and are available at the NCBI under the project accession numbers PRJNA590493, PRJNA658980, and PRJNA659050, respectively. For correspondence, the list of samples from previous datasets used in the present study is provided in Table S1. The data coming from different sequencing runs have been obtained and sequenced using the same protocols, and further processed together within a unique bioinformatic batch run.

### Metabarcoding data analysis

The 21 134 495 reads obtained from the 200 samples were processed using the open-source mothur software (version 1.47.0) (Schloss et al. 2009). Reverse and forward sequences were assembled and trimmed to keep only sequences comprised between 385 and 396 nucleotides, containing less than eight homopolymers and no ambiguous base. The sequences were aligned using the 16S rRNA gene reading frame from SILVA 138.1 database (Quast et al. 2012). Chimeras were detected and removed with Uchime (Edgar et al. 2011) implemented in mothur. Processed sequences were clustered into operational taxonomic units (OTUs) with a similarity threshold of 97%. The taxonomic affiliation was carried out using the SILVA 138.1 database (Quast et al. 2012). Sequences affiliated to chloroplasts, mitochondria, or eukaryotes were removed with the *remove.lineage* function in mothur. The singletons and OTUs with a relative abundance <0.005% were removed as recommended by Bokulich et al. (2013), leading to 1247 OTUs and 7274 315 sequences. Samples were rarefied to 6000 sequences with the *rarefy_even_depth function* (*phyloseq* package, version 1.42.0) (McMurdie and Holmes 2013), leading to the removal of three samples with lower sequence counts. This approach was used to normalize the dataset to a similar sequencing depth per sample, even though rarefying only once could lead to the misrepresentation of rare taxa that could be avoided by permutational rarefaction approaches (Schloss 2024).

A phylogenetic tree was built based on the aligned representative sequence of each OTU, using FastTree software with approximately maximum-likelihood estimation method and a generalized time-reversible model of nucleotide evolution.

### Bacterial community diversity and structure

The analyses were conducted from the rarefied OTU table in R version 4.3.2. The Shannon alpha diversity index was calculated using the *estimate_richness* function from the *phyloseq* package. Faith’s phylogenetic diversity (PD) index was calculated using *estimate_pd* function from *btools* package (version 0.0.1) for each habitat. The proportion of generalist and specialist OTUs in each habitat was assessed through the Levin’s niche breadth index (Bj) (Levins 1968) calculated with *levins.Bn* function from *MicroNiche* package (version 1.0.0) (Finn et al. 2020). A high Bj index indicates that the OTU is evenly distributed across the samples, signifying a generalist trait. Conversely, a low Bj index suggests that the OTU is present in a smaller proportion of the samples, indicative of a specialist trait. The community variability within each habitat was evaluated by an analysis of multivariate homogeneity of groups’ dispersions (*betadisper* function, *vegan* package).

Statistical comparisons of the Bj indexes between habitats, of the diversity indices among habitats and among sites (for a given habitat), and of the community variability between habitats were assessed using a nonparametric Kruskal–Wallis test (*kruskal.test* function) followed by a *post hoc* Dunn test with Holm correction (*dunn.test* function, dunn.test package version 1.3.5).

The Bray–Curtis and Jaccard (presence–absence) dissimilarity matrices were calculated from the rarefied OTU table, and the unweighted UniFrac dissimilarity matrix was calculated from the phylogenetic tree, using the *distance* function in the *phyloseq* package. To assess the effect of site, habitat, and host species on bacterial structure, a permutational multivariate analysis of variance (PERMANOVA) was conducted on the three dissimilarity matrices, using the *adonis2* and *pairwise.adonis* functions with Holm’s *P*-value correction to account for multiple comparisons. The Bray–Curtis dissimilarity matrix was used to perform principal coordinates analyses (PCoA) with the *ordinate* and *plot_ordination* functions from *phyloseq* package, either for all samples together or for each habitat separately.

To identify the OTUs that are significantly enriched in each habitat, a linear discriminant analysis effect size (LEFse, *run_lefse* function, *microbiomeMarker* package version 1.8.0) was performed with 100 bootstraps and the LDA score cut off by default (2). To visualize the relative abundance (per sample) of the ten most discriminant OTUs per habitat, a heatmap was plotted (*pheatmap* function *pheatmap* package version 1.0.12).

### Relationship between bacterial community, environment, and geography

To assess the relative effect of geographic distance and environmental differences on the community assemblages in each habitat, a variation partitioning (*varpart* function, *vegan* package) was performed with the Bray–Curtis dissimilarity matrix (bacterial community), and the PCNM variables and the PCA-based environmental variables as explanatory factors. The significance of each explanatory factor was tested by a permutation test (*anova.cca* function *vegan* package with 999 permutations).

Two distance–decay relationships (DDR) were calculated for each habitat, either (i) between the Bray–Curtis dissimilarity and the (log-transformed) pairwise geographic distance, or (ii) between the Bray–Curtis dissimilarity and the (log-transformed) pairwise environmental distance matrices. To statistically test the difference in DDR slope steepness among habitats, 1000 random resamplings of 21 samples for each habitat (which corresponds to 75% of the samples in the smallest dataset) were performed and DDR were computed for each bootstrap. The resulting linear model slopes of the DDR were compared between habitats with a Wilcoxon test.

### Quantification of bacterial community ecological assembly processes

The relative contribution of stochastic (i.e. ecological drift and dispersal limitation) and deterministic (i.e. homogeneous and variable selection) assembly processes was estimated for each habitat, within and among sites, using the analytical framework developed by Stegen et al. (2013). This method is based on the turnover of the phylogenetic and OTU composition. First, for a given habitat, the phylogenetic turnover of bacterial communities between two samples (either within or among sites) is calculated, to get the β mean nearest-taxon distance (βMNTD). The phylogenetic turnover was compared to a random null model expectation, and the difference between measured βMNTD and the mean of the null βMNTD distribution is referred to as the β-nearest taxon index (βNTI). Absolute βNTI values superior to |2| indicates that the observed turnover between a pair of communities is governed primarily by selection (specifically, by variable selection for βNTI > 2, and by homogeneous selection for βNTI <–2). A βNTI value between −2 and 2 indicates that the community is governed by stochastic processes (drift or dispersal). In such case, the turnover in OTU composition is further estimated using the pairwise Bray–Curtis based Raup–Crick dissimilarity index (RC_Bray_) between pairs of samples of a given habitat, either within or among sites. RC_Bray_ values inferior to –0.95 and superior to 0.95 correspond to communities that have more or fewer taxa in common than expected by chance, respectively, and therefore indicate that community turnover is driven by homogenizing dispersal (RC_Bray_ < –0.95) or dispersal limitation (RC_Bray_ > 0.95). On the contrary, RC_Bray_ values between –0.95 and 0.95 are indicative of ecological drift. The calculation of the βNTI and RC_Bray_ matrices was performed using an optimized version of the *ses.comdistnt* function, *MicEvo* package (version 0.9.19) developed by Richter-Heitmann et al. (2020).

## Results

### Environmental characterization of each site

The PCA of the environmental variables revealed a clear latitudinal separation along the first axis (explaining 74% of the observed variance) between the Antarctic site (South Shetland Islands), the Subantarctic islands, and Patagonia (Fig. 1B). South Shetland and South Georgia sites were more correlated with the oxygen and silicate concentrations, while the Patagonia site was more correlated with iron concentration and light availability. The Falkland/Malvinas site was correlated with pH, temperature, and primary production indicators (such as chlorophyll a concentration, primary production, and carbon phytoplankton biomass), whereas the Kerguelen site was more correlated with current velocity (Fig. 1B).

### The *Abatus* model provides a gradient of habitat specificity for bacterial communities

**Figure 2.**
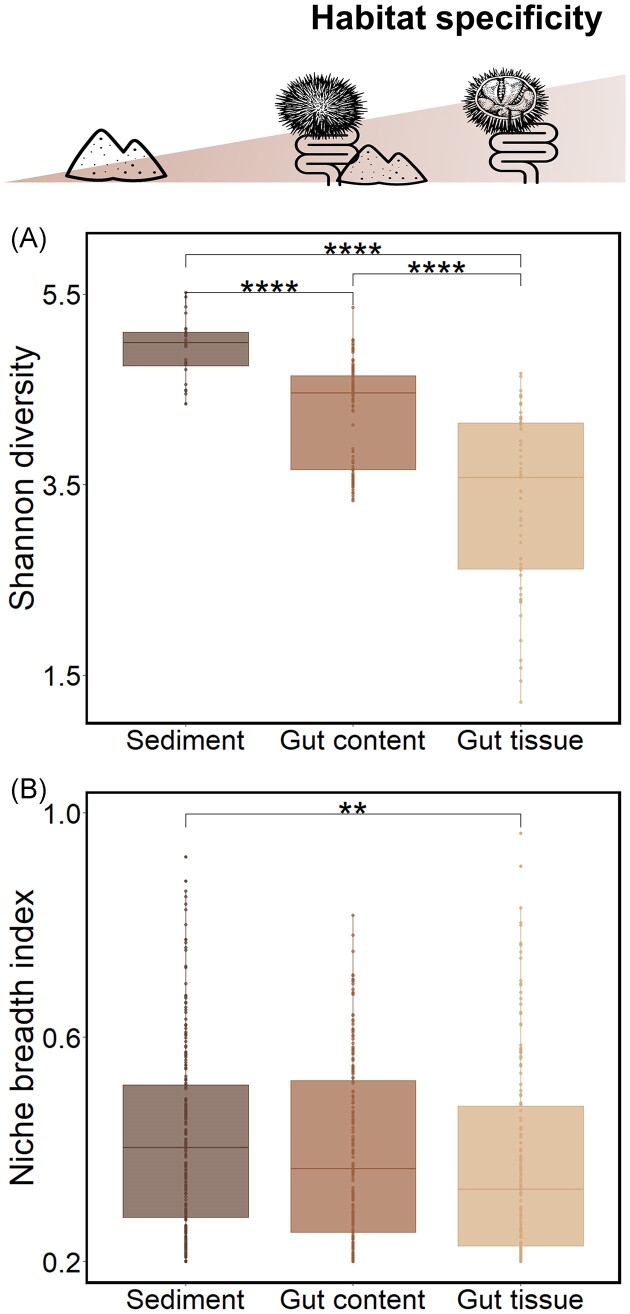
Characterization of the habitat specificity gradient. (A) Boxplot of alpha diversity estimated by the Shannon index for each habitat (sediment, *n* = 28; gut content, *n* = 110; and gut tissue, *n* = 59). (B) Boxplot of Levins niche breadth index (Bj) for each habitat. Only significant differences among habitats are shown by brackets (Kruskal–Wallis, followed by *post hoc* Dunn test). The significance level of *P*-values is indicated as follows: * < .05, ** < .01, *** < .001, and ^****^ < .0001.

A gradient of alpha diversity was observed across the three habitats, with significantly higher values of Shannon index for the sediment than for the gut tissue bacterial communities (*P*-value < .001; Fig. 2A). The same pattern was observed for the PD index (Fig. S1), suggesting that OTUs from the gut tissue are phylogenetically more related to each other than the ones from the sediment. The sediment OTUs had significantly higher niche breadth index than the OTUs from gut tissue habitats (Fig. 2B), indicating that sediment has more generalist OTUs while gut tissue OTUs are more specialists. The gradients of alpha diversity and niche breadth index were consistent and more pronounced within each locality considered independently (Fig. S2).

### The site differentiation decreases along the gradient of increasing habitat specificity

**Figure 3.**
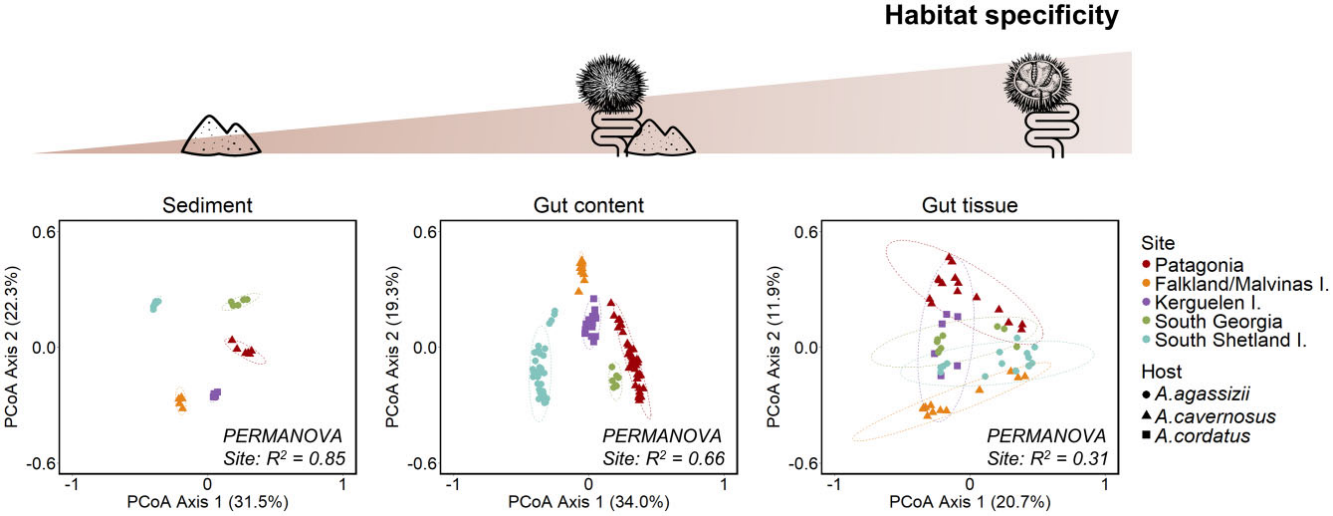
Dissimilarity of bacterial communities along the gradient of habitat specificity. PCoA of bacterial community composition based on Bray–Curtis distance from 16S rRNA gene sequences, for each habitat separately. Symbol colours represent sampling sites, and symbol shapes indicate the host species. Ellipses represent the standard deviation of all points for a given site with a confidence interval at 0.95. The effect of the site on the clustering was tested by PERMANOVA (*adonis2* function *vegan* package, all *P*-values < .05).

For the three habitats, the sampling site was the strongest driver of community variation (PERMANOVA, *P*-values < .001; Table S2). However, the effect of the sampling sites was weaker for more specific habitats (gut tissue) than for external sediment. The magnitude of the site effect thus decreased along the gradient of increasing habitat specificity. The highest divergence among sites was observed for the sediment bacterial communities (*R*^2^ = 0.85), followed by the gut content bacterial communities (*R*^2^ = 0.66) and the gut tissue bacterial communities (*R*^2^ = 0.31) (Fig. 3; Table S2). For the sediment and gut content bacterial communities, each site formed a separated cluster on the PCoA, and the first axis (explaining >30% of the variation) mainly separated the South Shetland site from the Subantarctic sites (Fig. 3). Contrary to what was expected from the biogeographic provinces and host distribution, the bacterial communities from South Georgia clustered closer to the Subantarctic sites than to the Antarctic one.

By contrast, the gut tissue bacterial communities were not clearly separated by sampling site in the PCoA ordination (Fig. 3). This can be due to the variability of the gut tissue communities among replicates, which was significantly higher than for gut content and sediment samples (as shown by *betadisper* analysis; Fig. S3), independently of the distance matrix used (Table S3). The decrease in the site differentiation along the gradient of habitat specificity was consistent when analysing Bray–Curtis, Jaccard, or unweighted UniFrac dissimilarity matrices. The magnitude of the site effect (indicated by the PERMANOVA *R*^2^ value) was lower for Jaccard and unweighted UniFrac distances than for Bray–Curtis (Table S2). Pairwise differences between sites did not show a common pattern similar for the three habitats (Table S4). For example, for the sediment, the most different bacterial communities were observed between South Shetland and Kerguelen (*R*^2^ = 0.90; Table S4), while for the gut tissue the most different bacterial communities were observed between Kerguelen and South Georgia (*R*^2^ = 0.28; Table S4).

### The contributions of environmental and geographic factors to bacterial community variation decrease along the gradient of increasing habitat specificity

**Figure 4.**
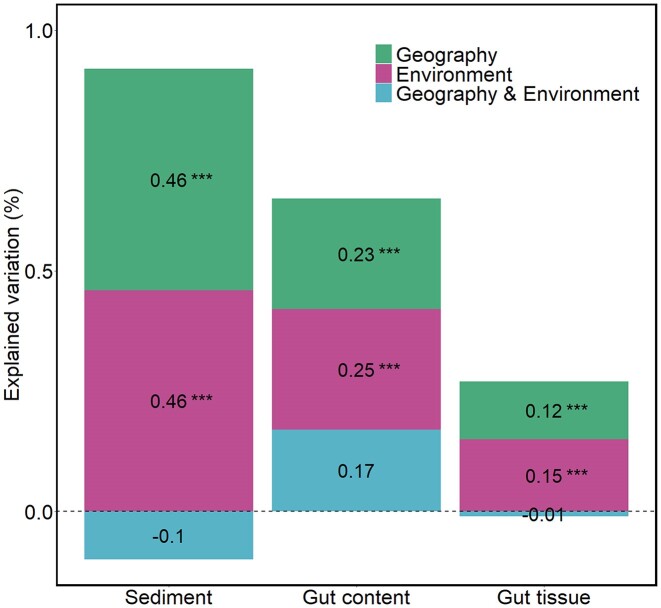
Variation partitioning analysis showing the amount of variation explained by geography and environment on bacterial community dissimilarity for each habitat. The bacterial dissimilarity matrix was calculated with Bray–Curtis. The geographic distance matrix was obtained from the three principal coordinates of a neighbourhood matrix (PCNM1 and PCNM2), and the environmental distance matrix was obtained from the sample scores on the two first principal components (PC1 and PC2) of the PCA of environmental variables. The significance of each factor was tested by 999 permutations and for each habitat separately (*anova.cca* function, *vegan* package) (Table S6).

The site effect is an integrative factor that combines the potential effects of geographical distance and environmental differences among sites. Variation partitioning analysis showed that geography and environment explained similar amounts of variance in all habitats (Fig. 4). The amount of variance explained by geography and environment decreased along the gradient of habitat specificity, being two to three times lower in gut tissue bacterial communities than in sediment bacterial communities (Fig. 4). When normalized by the total amount of explained variation, the relative contribution of geography and environment remained stable (representing around 50% each) along the gradient of habitat specificity.

**Figure 5.**
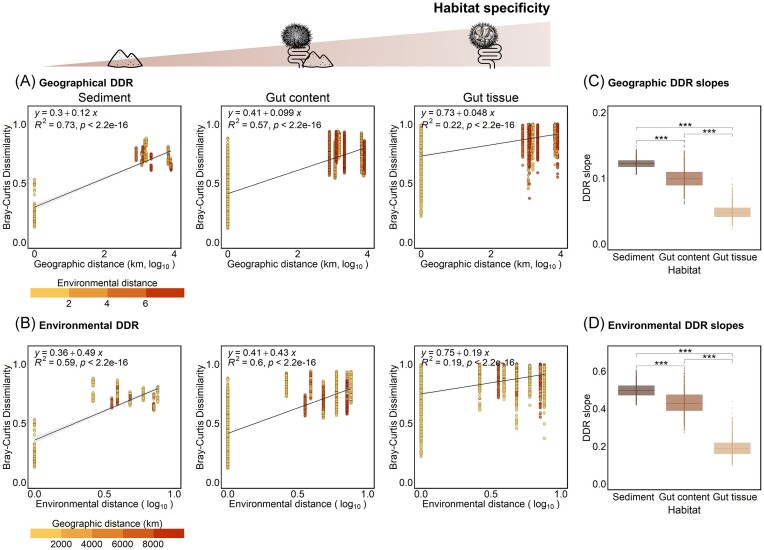
DDR along the gradient of habitat specificity. (A) DDR of bacterial communities’ dissimilarity (calculated as Bray–Curtis dissimilarity) according to the geographic distance for each habitat. Dot colours represent the environmental distances between samples calculated from the Euclidean distance matrix of environmental values. (B) DDR of bacterial communities’ dissimilarity (calculated as Bray–Curtis dissimilarity) according to the environmental distance for each habitat. Dot colours represent the geographic distances (km) between samples. Linear regressions were added on each scatterplot, together with the equation, the coefficient of determination (*R*^2^), and the *P*-value of the regression model (p). Geographical and environmental scales were log_10_ transformed. (C and D) Distribution of geographical (C) and environmental (D) DDR slopes for each habitat, obtained from 1000 bootstraps. Only significant differences among habitats are shown by brackets (Wilcoxon test). The significance level of *P*-values is indicated as follow: * < .05, ** < .01, *** < .001, and ^****^ < .0001.

DDR were established between community dissimilarity and either the geographical (Fig. 5A) or environmental (Fig. 5B) distances for each habitat. The slopes of the geographical and environmental DDR were significantly higher for sediment (*R* = 0.12 and 0.49, respectively) than for gut tissue bacterial communities (*R* = 0.05 and 0.19, respectively, Wilcoxon test, *P*-values < .001; Fig. 5C and D), indicating that the strength of the geography and environment effects decreased along the gradient of increasing habitat specificity. This result suggests a stronger geographical and environmental divergence for less selective habitats such as sediment compared to gut tissue habitat.

### Habitat-specific taxa are distributed along the gradient of habitat specificity

**Figure 6.**
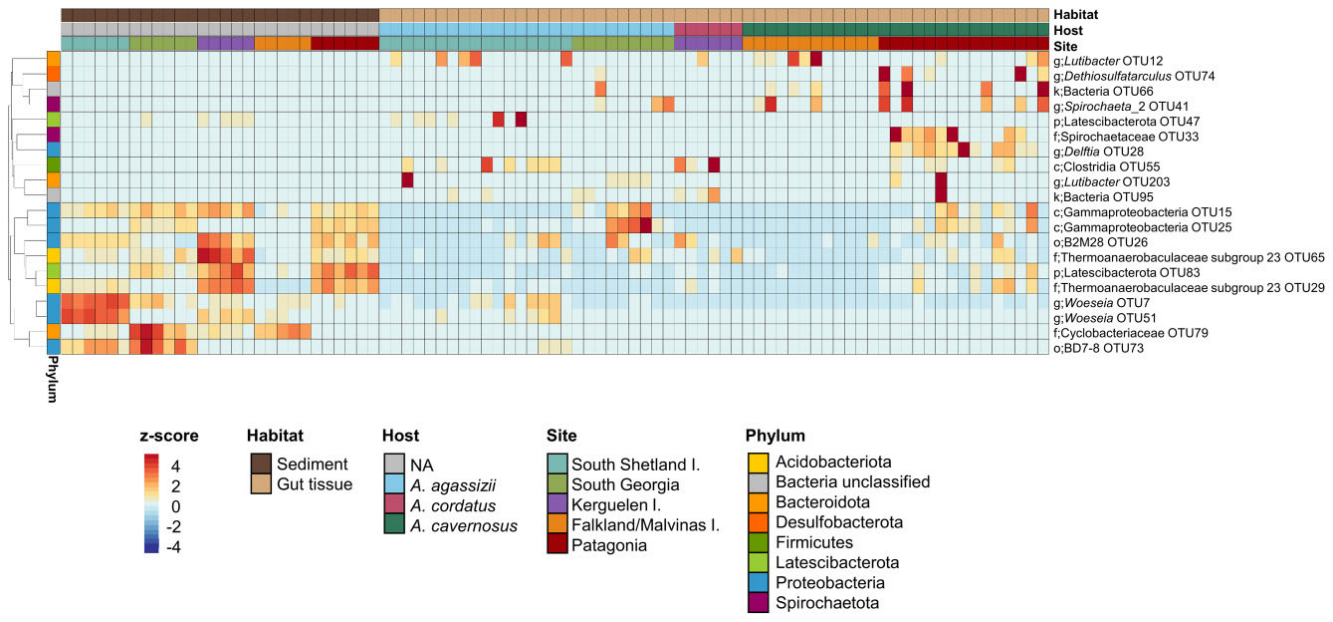
Sediment and gut tissue discriminant OTUs. Heatmap illustrating the z-score distribution of the top 10 most discriminant OTUs (LEFse analysis, LDA score > 2, *P*-value < .01, Wilcoxon rank-sum test) for sediment and gut tissue bacterial communities. OTUs are annotated with their best taxonomic affiliation. Letters before the taxon name indicate the taxonomic level: k (Kingdom), p (Phylum), c (Class), o (Order), f (Family), and g (Genus). For clarity purpose, only sediment and gut content are presented here, the discriminant OTUs of the gut content habitat are shown in Table S7.

Each habitat was characterized by specifically enriched OTUs. Sediment bacterial communities were characterized by OTUs mainly belonging to Proteobacteria and Acidobacteria phyla (Fig. 6). Only 2.1% of the sediment OTUs were shared among all samples, and 19% of the sediment OTUs were shared among the five sites (but were not necessarily present in all samples of each site) (Fig. S4A). The main discriminant sediment-enriched OTUs differed according to the site of provenance: *Woeseia* for South Shetland Islands*, Cyclobacteriaceae* for South Georgia and Falkland/Malvinas Islands, *Thermoanaerobaculaceae* for Kerguelen Islands, Gammaproteobacteria BD7-8 for South Georgia, and Latescibacterota for Patagonia (Fig. 6). Gut content bacterial communities were characterized by OTUs belonging to Planctomycetota and Proteobacteria phyla in particularly *Blastopirellula* and *Rubripirellula* genera, *Rhodobacteraceae* family and Gammaproteobacteria class (Table S6). Gut tissue bacterial communities were characterized by OTUs belonging to more diverse phyla (Table S6 and Fig. 6). None of the gut tissue OTUs was shared among all samples, and only 21% were shared among the three *Abatus* species (Fig. S5A). Contrary to what was observed in sediment, the gut tissue discriminant taxa did not show a site-specific pattern. The gut tissue discriminant OTUs could be either significantly enriched in one of the sea urchin species, such as *Dethiosulfatarculus* and *Delftia* (in *A. cavernosus*), Latescibacterota (in *A. agassizii*), Clostridia (in *A. cordatus*), or in several host species such as *Lutibacter* and *Spirochaetaceae* (Fig. 6). A more detailed list of OTUs significantly enriched in the sediment and gut tissue is provided in Figs S4 and S5.

### Ecological processes governing bacterial community assembly differ along the gradient of habitat specificity

**Figure 7.**
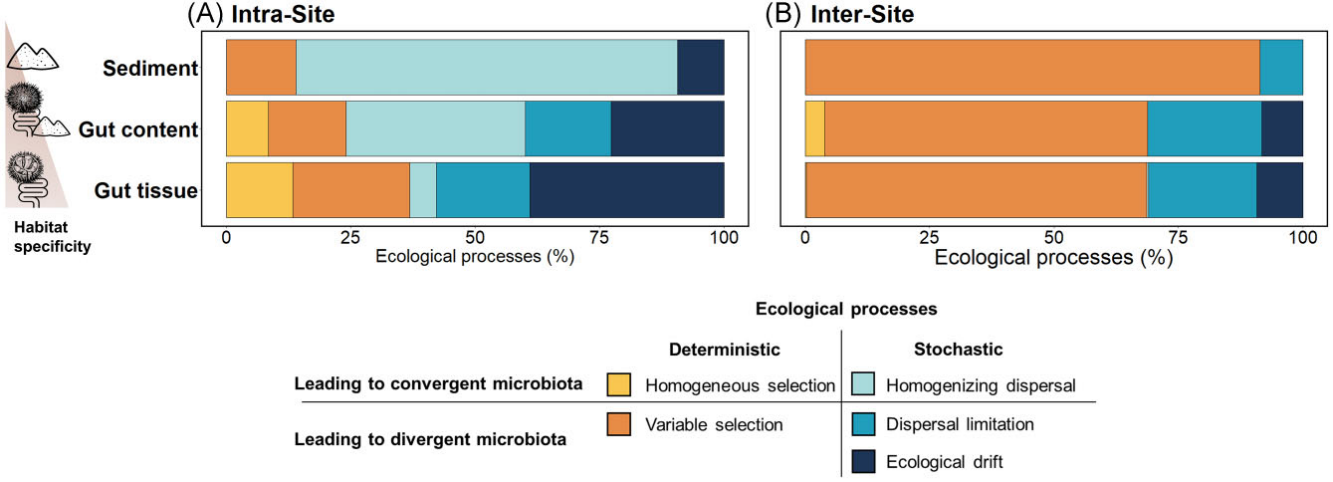
Ecological processes driving the assembly and shift of bacterial communities in each habitat. Relative importance of each assembly process governing the bacterial communities’ assembly within sites (intrasite, A) and among sites (intersite, B) for each habitat. For detailed percentage of each ecological process contribution see Table S8 and for statistical differences between habitat see Table S9.

Within each site, stochastic processes were the dominant assembly mechanisms in the three habitats. Their contribution significantly decreased along the gradient of habitat specificity representing 86.0%, 65.8%, and 63.0% for sediment, gut content, and gut tissue, respectively (Fig. 7; Tables S7 and S8). The nature of the stochastic processes also differed strikingly between sediment bacterial communities [dominated by homogenizing dispersal (76.7%)] and gut tissue bacterial communities [dominated by ecological drift (38.9%) and dispersal limitation (18.8%)] (Fig. 7; Table S7). These results suggest that the sediment bacterial communities are more connected and can easily disperse at a small space scale, allowing homogenization within the same site, while gut tissue bacterial communities had lower dispersal ability and were characterized by more variability and random colonization. By contrast, the contribution of deterministic processes significantly increased along the gradient of increasing habitat specificity from 14.0% to 24.1% and 36.8% for sediment, gut content, and gut tissue, respectively (Fig. 7; Tables S7 and S8). Among these deterministic processes, the contribution of variable selection did not significantly differ between habitats (Table S8), while homogenous selection only contributed to the community assembly of gut-related habitats (8.5% and 13.4% for gut content and gut tissue, respectively), suggesting that the intestinal conditions, likely similar for individuals of the same species, might select similar bacterial taxa, thus homogenizing part of the community.

When comparing different sites, the main process driving bacterial community assembly was variable selection for all habitats (>64.8%; Fig. 7; Table S7) indicating that the differences of environment among sites imposed differential species sorting, thus driving differences in bacterial communities’ composition across sites. The relative contribution of variable selection decreased along the gradient of increasing habitat selectivity, from 91.3% for sediment to 68.3% for gut tissue bacterial communities (Fig. 7; Table S7), suggesting that the gut tissue bacterial communities are constrained in a lesser extent by the external environmental conditions among sites than sediment bacterial communities. Stochastic processes, mainly dispersal limitation and, to a lesser extent, ecological drift, contributed about 30% (Fig. 7; Table S7) to the assembly of bacterial communities from gut content and gut tissue, indicating that random processes are also important in structuring gut bacterial communities between different sites.

## Discussion

### The *Abatus* gut acts as a ‘specific habitat’ for bacterial communities

The difference between gut microbiota and the surrounding environment has been observed in various sea urchins (Hakim et al. 2016, 2019, Rodríguez-Barreras et al. 2021). The reduction of taxonomic and phylogenetic bacterial diversity along the gradient of habitat specificity imposed by *Abatus* gut, previously observed for a single species and site (Schwob et al. 2020), was expanded here to three species and five sampling sites across the Southern Ocean. The microbiota of *Abatus* gut tissue harbours a higher proportion of specialist taxa than in the surrounding sediment. This result could be due to a shared evolutionary history between the host and its bacterial symbionts (i.e. coevolution or cophylogeny; Moran and Sloan 2015, Pankey et al. 2022, Schwob et al. 2024) or may reflect a phylosymbiosis pattern, as evidenced in other sea urchins (Carrier et al. 2024). Gut tissue microbiota presented a high individual variability, as also evidenced in other marine organisms such as sponges (Griffiths et al. 2019), fishes (Stagaman et al. 2017), and copepods (Datta et al. 2018). Such variability could be due to host intrinsic related factors (immunology and genetics), microbial interactions, and/or stochastic processes that could vary between individuals (Adair and Douglas 2017). Despite the interindividual variability, OTUs from *Lutibacter, Dethiosulfatarculus, Spirochaeta-2, Delftia*, and *Spirochaetaceae* were significantly enriched in the *Abatus* gut tissue from all species and sites. *Spirochaetaceae* have already been identified as keystone in the core gut microbiota of *A. agassizii* (Schwob et al. 2020) and other sea urchins from the Schizasterid family (Ziegler et al. 2020), and display phylogeographic patterns at intra-OTU level (i.e. microdiversity) (Schwob et al. 2021). A *Lutibacter* strain has been isolated from the sea urchin *Strongylocentrotus intermedius* (Nedashkovskaya et al. 2015). Bacterial taxa that are conserved over time and space among our three ecologically and phylogenetically related sea urchin species (Díaz et al. 2012) might be important for host functioning (Murfin et al. 2015, Brooks et al. 2016). For instance, *Dethiosulfatarculus* and *Delftia* genera comprise, respectively, anaerobic sulphate reducers (Davidova et al. 2016) and aerobic, nonfermentative, nitrate reducers (Wen et al. 1999), that might contribute to host nutrition via nitrate and sulphate metabolisms. In Antarctic organisms, such as sponges, exclusive bacterial symbionts display functions not only related to nutrient metabolism, but also enabling their symbiotic lifestyle and adaptation to cold environments (Moreno-Pino et al. 2024).

### Strong biogeographical pattern in sediment bacterial communities across the Southern Ocean

The sediment bacterial communities exhibited the strongest biogeographical clustering pattern across the five sites in the Southern Ocean, with a notable contrast between the Antarctic and Subantarctic provinces. This contrast could be explained by the APF that promotes physicochemical and biological dissimilarities between Antarctic and Subantarctic regions and acts as a strong biogeographic barrier, as mainly documented for free-living surface seawater communities to date (Wilkins et al. 2013, Wang et al. 2020, Maturana-Martínez et al. 2022, Sow et al. 2022), as well as for *Spirochaeta* microdiversity (phylotypes) in *Abatus* gut tissue (Schwob et al. 2021). Biogeographic studies of Antarctic benthic environments are scarcer and focus on deep sea sediment (Li et al. 2020), or intertidal sediments at a small geographical scale (Wang et al. 2017). Comparisons between Antarctic and Subantarctic marine sediment bacterial communities do not address biogeographic concerns (Espínola et al. 2018, Galván et al. 2023). The sediment-discriminating taxa are discussed in Supplementary Material S1.

The biogeographical classification of South Georgia biota (Antarctic versus subAntarctic) is still debated and is taxa-dependent (Supplementary Material S2). Here, contrary to the host phylogeographic pattern, the sediment bacterial communities from South Georgia showed the highest dissimilarity with the Antarctic ones and clustered more closely with the subAntarctic communities (Patagonia and Falkland/Malvinas Islands). This result can be explained by the eastward dispersal of sediment bacteria promoted by the ACC, as previously suggested for other sites of the Southern Ocean (Schwob et al. 2021), since the southern boundary of the ACC is located south of South Georgia (Thorpe et al. 2002).

The differentiation of sediment bacterial communities among sites was explained by a similar level of contribution of geographic and environmental distances, in contrast with most studies highlighting a predominant role of local factors (i.e. environmental selection) in structuring sediment and soil bacterial communities (Wang et al. 2017, Hoshino et al. 2020, Maturana-Martínez et al. 2022, Seppey et al. 2023). Our results are consistent with the patterns observed for deep-sea sediment around Antarctica (Li et al. 2020) and in the South Atlantic Ocean (Schauer et al. 2010), as well as for continuous, well-connected Southern Ocean surface water (Wang et al. 2020), thus expanding these findings at larger scale across different biogeographic provinces.

### The strength of the biogeographical pattern is modulated by the level of habitat specificity

The differentiation of bacterial communities between sites decreased along the increasing gradient of habitat specificity. The effect of environmental and geographical distances on bacterial community variation was stronger for sediment (less specific habitat) than for gut tissue (more specific habitat). Similarly, steeper DDR based on geographic and environmental distances have been previously evidenced in nonhost-associated marine habitats characterized by a higher environmental heterogeneity (Zinger et al. 2014, Zhao et al. 2022). Even if contrary results have been reported at a smaller spatial scale in terrestrial ecosystem (soil versus earthworm gut), the DDR strength decreased from the earthworm foregut to the hindgut, suggesting that bacterial community became more similar during the passage of the gut, thus reflecting a potential gradient of habitat specificity within the intestinal tract (Wu et al. 2023). Our results are consistent with the conceptual framework proposed by (Langenheder and Lindström 2019), where more heterogenous habitats, represented by marine sediment in our case, are expected to be more impacted by environmental filtering. This can be attributed to the large differences in environmental conditions of sediment among sites, whereas the *Abatus* gut conditions might homogenize the local environment among sites and select specific bacterial taxa, thus reducing the influence of external factors and environmental heterogeneity to which sea urchins are exposed. When comparing free-living and host-associated bacterial communities across six Australian estuaries, the bacterial communities from less selective and more heterogenous habitats (seawater and sediment) showed a stronger correlation with geographic and environmental factors compared to the fish-associated ones (Suzzi et al. 2023), similarly to our conclusions. This suggests that other factors, likely related to the host (e.g. physicochemical conditions of the gut, immunity, genetics, physiology, diet, microbial interactions, among others—not measured in this study), might play a crucial role in structuring the gut microbiota (Adair and Douglas 2017, Xiao et al. 2021, Kang et al. 2022, Pan et al. 2022), potentially overwhelming the geographic and environmental effects and promoting the homogenization of the gut bacterial communities. Host species or population genetics have been demonstrated as significant factors structuring bacterial community assembly (Pita et al. 2013, Easson et al. 2020, Sacristán-Soriano et al. 2020). In addition to ecological factors, the evolution of the holobiont can also be the result of cophylogeny and codiversification processes, where both the host and its associated microbiota coevolve over time alongside environmental convergence (Pankey et al. 2022).

Here, the host- and site-effects could be partly confounded due to the absence of different *Abatus* species coexisting in the same sites. In other words, the fact that each site harbours only one *Abatus* species make it challenging to confidently attribute community variability to either a site or a host effect. However, our study design includes two *Abatus* species that are present at different sites: (i) *A. cavernosus* in Patagonia and Falkland; (ii) *A. agassizii* in South Shetland and South Georgia. Thus, the observed differences of gut microbial communities between these sites (i.e. Patagonia versus Falkland on the one hand; South Shetland versus South Georgia on the other hand) are likely site-driven rather than host-driven.

Apart from environmental and spatial factors, we cannot exclude the role of vertical transmission (from parents to offsprings) in structuring the gut microbiota of several invertebrates (Sharp et al. 2007, Baldassarre et al. 2021), including sea urchins (Carrier et al. 2021). However, in most cases microbiota acquisition is governed by a combination of vertical and horizontal transmission (from the environment) (Sipkema et al. 2015, Morrow et al. 2018, Björk et al. 2019, de Oliveira et al. 2020, Giraud et al. 2022, Unzueta-Martínez et al. 2022, Buschi et al. 2023). Horizontal transmission from feeding has been recognized in echinoderms larvae (Carrier and Reitzel 2020, Carrier et al. 2020). Regardless of the acquisition route, the gut microbiota is expected to change throughout an individuals’ lifespan in tight relation with the environment and diet (Carrier et al. 2021, Masasa et al. 2021, Renelies-Hamilton et al. 2021, Kang et al. 2022). In our study, several evidence challenge the hypothesis of exclusive vertical transmission of *Abatus* gut microbiota. First, a large number of taxa were shared between the *Abatus* gut tissue and the external sediment communities (1080 common OTUs out of 1172 OTUs in gut tissue; Fig. S7), accounting for 90% of the abundance of the gut tissue community (Table S9). Second, despite multiple attempts, we were unable to detect a microbial signal in *Abatus* eggs and gastrulas through PCR amplification (see Supplementary methods, Fig. S8, Table S10). Third, the assembly of the gut bacterial communities was primarily driven by stochastic processes (Fig. 7A), whereas homogeneous selection would be expected to dominate if vertical transmission were the sole mode of microbiota acquisition—implying the consistent transmission of obligate symbionts across generations. Fourth, we did not identify OTUs common to all *Abatus* gut samples, even at the scale of one *Abatus* species nor of one site (Fig. 6; Fig. S5), which is contradictory with the vertical transmission and maintenance of important bacterial symbionts. Therefore, even if a fraction of the gut microbiota is vertically transmitted, it might be marginal compared to the community variability induced by geographic and environmental factors.

### The contribution of ecological processes to the bacterial assembly is modulated by the degree of habitat specificity and the geographical scale

At a local scale (i.e. within each site), stochastic processes dominated the assembly of bacterial communities in the three habitats. The relative contribution of stochastic processes decreased along the increasing gradient of habitat specificity from sediment to gut tissue, consistently with Dickey et al. (2021). In soils, the contribution of assembly stochastic processes varies according to the habitat heterogeneity level, albeit in an opposite way for specialist versus generalist taxa (Gao et al. 2023). In our study, the main process governing the assembly of sediment bacterial communities was homogenizing dispersal, probably due to the higher connection between sediment communities than between gut communities. Indeed, in our coastal sampling sites, the semidiurnal tides, and the bioturbation activity of *Abatus* sea urchins (Lohrer et al. 2005, Thompson and Riddle 2005) can generate sediment resuspension and mixing, facilitating the dispersion of bacteria within the same locality. On the other hand, the gut of different *Abatus* individuals coming from the same site can be considered as island-like habitats (Loudon et al. 2016, Itescu 2019), thus limiting the dispersion capacity of gut bacteria from one host to another and explaining the contribution of dispersal limitation in this habitat. By contrast, the main assembly process for gut tissue bacterial communities was ecological drift, as also observed in zebrafish (Xiao et al. 2021). Bacterial colonization of the gut could generate random assemblage due to the priority effect (Fukami 2015), since the initial colonizing bacterium will influence, through biological interactions, the subsequent establishment and maintenance of bacteria. Regarding the (minor) deterministic processes, homogenous selection was significantly more important in the most specific habitat (gut tissue, containing the highest fraction of specialists) suggesting that the filter imposed by hosts to their bacterial symbionts dictates some homogenizing patterns in gut community assembly common to all hosts. This is consistent with the fact that specialist taxa, being representative and well adapted to specific habitats, are generally more affected by deterministic factors than generalist taxa (Székely and Langenheder 2014, Liao et al. 2016). For instance, homogeneous selection has been demonstrated to be an important process in shaping fish gut tissue bacterial communities (Xiao et al. 2021). In contrast with our study, homogenous selection was the main process governing bacterial assembly in a highly connected Antarctic freshwater systems, especially in mats and slightly less in water, while dispersal limitation had little influence (Ramoneda et al. 2021) suggesting that the assembly mechanisms are dependent on the studied system and the geographical scale.

At a regional scale (i.e. among sites), the bacterial assemblage was primarily driven by variable selection in all habitats, with a significantly stronger contribution in sediment. The importance of variable selection in sediments can be explained by their higher environmental variability and heterogeneity among sites, selecting for different bacterial communities, as previously reported in other marine sediments (Petro et al. 2017). The proportion of variable selection was lower in gut tissue due to the homogenizing effect imposed by the specific gut conditions of closely related hosts; however it remains an important driver of community assembly, suggesting that the different host species found in the different sites might impose different gut filtering and select different communities. In parallel, the proportion of ecological drift and dispersal limitation increased in more specific habitats (gut tissue) among sites, probably due to less connected and island-like ecosystems, as evidenced in invertebrate gut bacterial communities (Sieber et al. 2019, Ge et al. 2021).

## Conclusion

Our results underscore the important role of habitat specificity in shaping bacterial biogeographic patterns in Southern Ocean benthic ecosystems. Locality, geography, and environmental factors exerted a greater influence on less specific habitats (i.e. sediment) than on more specific habitats (i.e. sea urchin gut tissue). Both stochastic and deterministic ecological processes played a potential role in bacterial assembly, with different magnitude according to the habitat specificity level and the geographical scale (i.e. within or among sites). Our study highlights the importance of considering diverse habitats when examining bacterial biogeographic patterns and their associated assembly processes. Understanding patterns of microbial diversity, which is fundamental, for example, for studying ecosystem functioning or for developing conservation strategies, requires nowadays a more in-depth evaluation of diverse habitats as they can behave very differently depending on their level of specificity.

## Supplementary Material

fiae134_Supplemental_Files

## Data Availability

Part of the dataset was extracted from previously published works and is available at the National Center for Biotechnology Information (NCBI) repository under the project accession numbers PRJNA590493, PRJNA658980 and PRJNA659050. Supplementary datasets generated for this study (Falkland/Malvinas and South Georgia samples) are also available at the NCBI repository under the accession number PRJNA1065352. The R codes used in this manuscript are publicly available on GitHub at https://github.com/MelanieDelleuze/Habitat_specificity_bacterial_biogeography_SO.git.
