# Supplementary material for: Habitat specificity modulates the bacterial biogeographic patterns in the Southern Ocean
Source: FEMS Microbiol Ecol. 2024 Oct 3;100(11):fiae134. doi: 10.1093/femsec/fiae134 (PMC11523047; doi:10.1093/femsec/fiae134)
Supplement: fiae134_Supplemental_Files [file fiae134_supplemental_files.zip › Supplementary_material_Delleuze_et_al_09_2024.docx]

**SUPPLEMENTARY MATERIAL**

**Habitat specificity modulates the bacterial biogeographic patterns in the Southern Ocean**

**Authors**

Mélanie Delleuze^1,2,3^, Guillaume Schwob^3^, Julieta Orlando^3,4^, Karin Gerard^3,5,6^, Thomas Saucède^7^, Paul Brickle^8,9^, Elie Poulin^1,3^, Léa Cabrol^3,10^

**Affiliations**

^1^Laboratorio de Ecología molecular, Departamento de Ciencias Ecológicas, Facultad de Ciencias, Universidad de Chile, Santiago, Chile

^2^Marine Biology Lab, CP160/15, Université Libre de Bruxelles (ULB), Brussels, Belgium

^3^Millennium Institute Biodiversity of Antarctic and Subantarctic Ecosystems (BASE), Santiago, Chile

^4^Laboratorio de Ecología Microbiana, Departamento de Ciencias Ecológicas, Facultad de Ciencias, Universidad de Chile, Santiago, Chile

^5^Laboratorio de Ecosistemas Marinos Antárticos y Subantárticos, Universidad de Magallanes, Punta Arenas, Chile

^6^Cape Horn Investigation Center, Puerto Williams, Chile

^7^Biogéosciences, UMR CNRS 6282, Université de Bourgogne, Dijon, France

^8^South Atlantic Environmental Research Institute, Stanley, Falkland Islands

^9^School of Biological Sciences (Zoology), University of Aberdeen, Aberdeen, Scotland, UK

^10^Mediterranean Institute of Oceanography, CNRS, IRD, M.I.O. UM 110, Aix-Marseille University, Univ Toulon, Marseille, France

**Table of Contents**

***Table S1:*** List of samples used from previous datasets (supplementary excel sheet)

***Figure S1:*** Faith’s Phylogenetic diversity along the gradient of habitat specificity3

***Figure S2:*** Alpha diversity and niche breadth along the gradient of habitat specificity for each sampling site.4

***Figure S3:*** Samples variability along the gradient of habitat specificity.5

***Table S2:*** Results of Multivariate permutational analysis of variance (PERMANOVA) 6

***Table S3:*** Analysis of multivariate homogeneity of groups’ dispersions (*betadisper*) 7

***Table S4:*** Results of Pairwise permanova comparisons among sites on bacterial community dissimilarity based on Bray-Curtis dissimilarity matrix for each habitat separately.8

***Table S5:*** Permutation test on the results of the variation partitioning analysis. 9

***Figure S4:*** Taxonomic differentiation of sediment microbiota10

***Figure S5:*** Taxonomic differentiation of *Abatus* spp gut tissue microbiota.11

***Table S6:*** Ten most discriminant OTUs for each habitat.12

***Table S7:*** Ecological processes driving the assembly and shift of bacterial communities in each habitat within and among sites13

***Table S8:*** Wilcoxon test between site means of the percentage of each assemblage process13

***Figure S6:*** Environmental and Geographical hierarchical clustering dendrogram analysis of sediment samples..14

***Figure S7:*** Venn diagram showing shared taxa between the three habitats…………………..………15

***Table S9:*** Number of OTUs and sequences shared between sediment and gut tissue habitat……….16

***Supplementary Methods S1***: DNA extraction and PCR amplification of eggs, gastrulas and juveniles supports the horizontal transmission hypothesis in *Abatus* sea urchins……………………………….17

***Table S10:*** Summary of DNA extraction method, DNA concentrations (ng/µl) and PCR amplification result for gut tissue, juveniles, eggs and gastrula samples of *Abatus* sea urchins…………………….17

***Figure S8:*** Picture of an electrophoresis gel showing the results of the V4-V5 16s rRNA gene PCR amplification for gut tissue, eggs, gastrula and juveniles samples……………………………………18

***Supplementary Material S1*** : Biogeographical distribution of sediment-specific OTUs 19

***Supplementary Material S2:*** Biogeographical classification of South Georgia biota………………..20


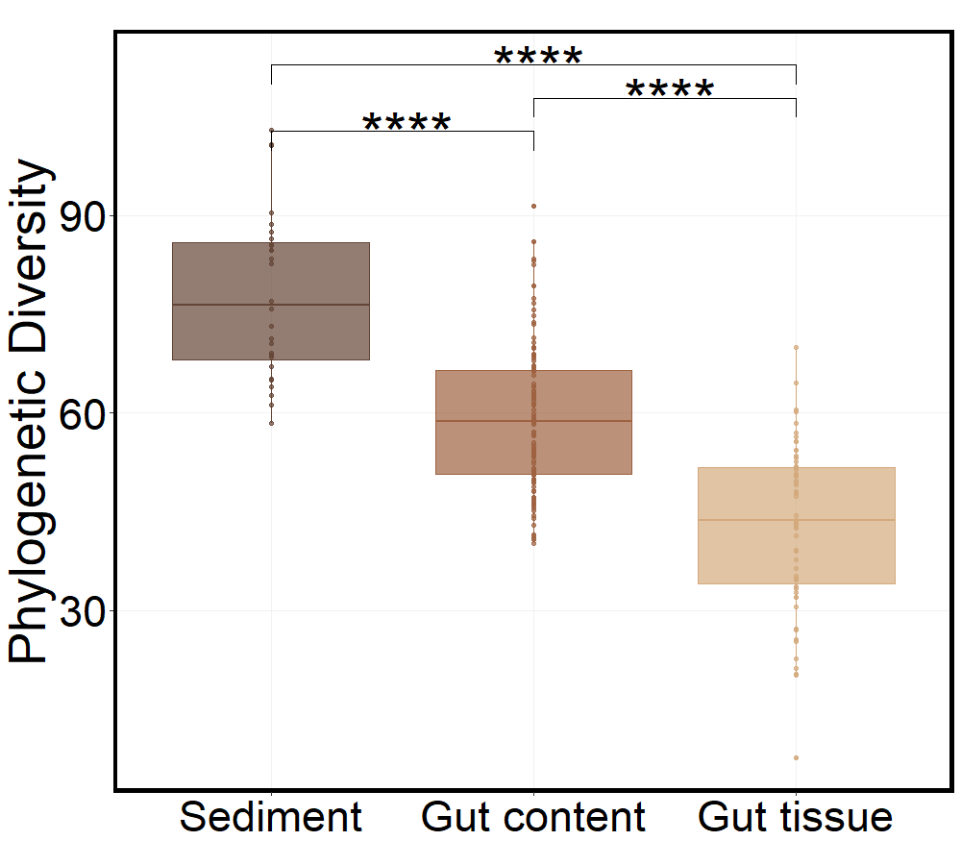


**Figure S1: Faith’s Phylogenetic diversity along the gradient of habitat specificity.** Boxplot of Faith’s Phylogenetic diversity (PD) index for each habitat. Only significant differences among habitats are shown by brackets (Kruskal-Wallis, followed by post-hoc Dunn test). The significance level of p-values are indicated as follow: * < 0.05, ** < 0.01, *** < 0.001, **** < 0.0001.


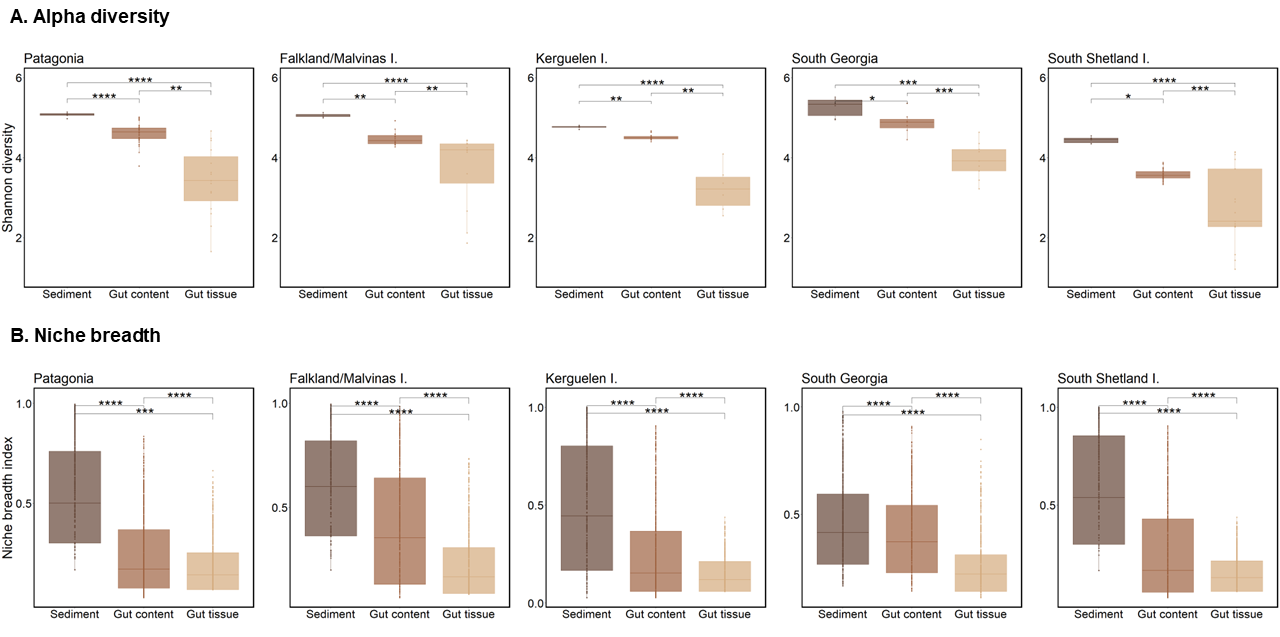


**Figure S2: Alpha diversity and niche breadth along the gradient of habitat specificity for each sampling site.** A) Boxplot of alpha diversity estimated by the Shannon index for each habitat in each site. B) Boxplot of Levins niche breadth index (Bj, representing the level of habitat generality) for each habitat in each site. Only significant differences among habitats are shown by brackets (Kruskal-Wallis, followed by post-hoc Dunn test). The significance level of p-values are indicated as follows: * < 0.05, ** < 0.01, *** < 0.001, **** < 0.0001.


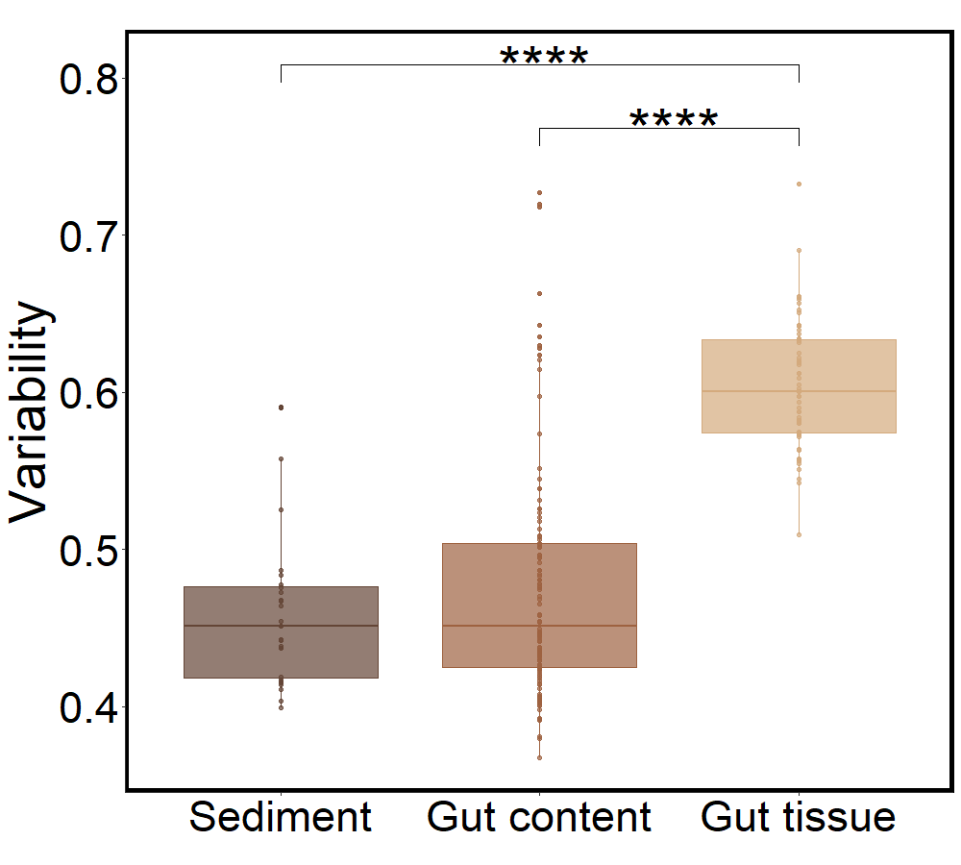


**Figure S3: Samples variability along the gradient of habitat specificity**. Boxplot of samples variability (*betadisper* analysis based on Bray-Curtis dissimilarity matrix) for each habitat, expressed as “distance to centroid”. Only significant differences among habitats are shown by brackets (Kruskal-Wallis, followed by post-hoc Dunn test). The significance level of p-values are indicated as follow: * < 0.05, ** < 0.01, *** < 0.001, **** < 0.0001.

**Table S2: Results of Multivariate permutational analysis of variance (PERMANOVA)** on bacterial community dissimilarity based on Bray-Curtis, Jaccard and unweighted UniFrac dissimilarity matrices for sediment, gut content and gut tissue habitats separately. “Site” corresponds to each sampling locality, “Host” corresponds to the *Abatus* species found in each of the sampling locality, and Province account for the division of Antarctic (including South Georgia and South Shetland I.) and Subantarctic (Patagonia, Falkland/Malvinas I., Kerguelen I.).

| Habitat | Distance matrix | Samples grouping | Df | Sums of square | F-statistics | R^2^ | P-value |
| --- | --- | --- | --- | --- | --- | --- | --- |
| Sediment | **Bray-Curtis** | **Site** | 4 | 5.12 | 32.63 | 0.85 | < 0.001 |
|  |  | **Host** | 2 | 2.16 | 7.02 | 0.36 | < 0.001 |
|  |  | **Province** | 1 | 1.32 | 7.36 | 0.22 | < 0.001 |
|  | **Jaccard** | **Site** | 4 | 6.22 | 17.84 | 0.76 | < 0.001 |
|  |  | **Host** | 2 | 2.80 | 6.47 | 0.34 | < 0.001 |
|  |  | **Province** | 1 | 1.60 | 6.31 | 0.19 | < 0.001 |
|  | **Unweigthed UniFrac** | **Site** | 4 | 2.3 | 13.4 | 0.70 | < 0.001 |
|  |  | **Host** | 2 | 0.96 | 5.19 | 0.29 | < 0.001 |
|  |  | **Province** | 1 | 0.54 | 5.16 | 0.17 | < 0.001 |
| Gut content | **Bray-Curtis** | **Site** | 4 | 16.75 | 50.19 | 0.66 | < 0.001 |
|  |  | **Host** | 2 | 9.16 | 29.94 | 0.36 | < 0.001 |
|  |  | **Province** | 1 | 6.36 | 35.83 | 0.25 | < 0.001 |
|  | **Jaccard** | **Site** | 4 | 18.23 | 30.17 | 0.53 | < 0.001 |
|  |  | **Host** | 2 | 10.47 | 23.69 | 0.31 | < 0.001 |
|  |  | **Province** | 1 | 6.91 | 27.46 | 0.20 | < 0.001 |
|  | **Unweigthed UniFrac** | **Site** | 4 | 8.29 | 29.2 | 0.53 | < 0.001 |
|  |  | **Host** | 2 | 4.64 | 22.36 | 0.29 | < 0.001 |
|  |  | **Province** | 1 | 3.40 | 28.85 | 0.22 | < 0.001 |
| Gut tissue | **Bray-Curtis** | **Site** | 4 | 6.65 | 5.99 | 0.31 | < 0.001 |
|  |  | **Host** | 2 | 2.72 | 4.02 | 0.13 | < 0.001 |
|  |  | **Province** | 1 | 1.70 | 4.88 | 0.08 | < 0.001 |
|  | **Jaccard** | **Site** | 4 | 5.77 | 4.17 | 0.24 | < 0.001 |
|  |  | **Host** | 2 | 2.39 | 3.04 | 0.098 | < 0.001 |
|  |  | **Province** | 1 | 1.47 | 3.65 | 0.06 | < 0.001 |
|  | **Unweigthed UniFrac** | **Site** | 4 | 4.67 | 7.74 | 0.36 | < 0.001 |
|  |  | **Host** | 2 | 2.05 | 5.35 | 0.16 | < 0.001 |
|  |  | **Province** | 1 | 1.32 | 6.56 | 0.10 | < 0.001 |

**Table S3: Analysis of multivariate homogeneity of groups’ dispersions (*betadisper*) results for the three distance matrices used (Bray-Curtis, Jaccard and unweighted UniFrac).** Statistical differences among habitats were tested with Kruskal-Wallis test (all p-values < 0.05) and followed by a Dunn post-hoc test with holm correction.

| **Distance matrix** | **Habitat** | **Average distance to median** | **Comparisons** | **Adjusted p-value** |
| --- | --- | --- | --- | --- |
| **Bray-Curtis** | Sediment | 0.46 | Sediment *vs* Gut content | 0.24 |
|  | Gut content | 0.48 | Gut content *vs* Gut tissue | <0.0001 |
|  | Gut tissue | 0.60 | Sediment *vs* Gut tissue | <0.0001 |
| **Jaccard** | Sediment | 0.54 | Sediment *vs* Gut content | 0.14 |
|  | Gut content | 0.55 | Gut content *vs* Gut tissue | <0.0001 |
|  | Gut tissue | 0.64 | Sediment *vs* Gut tissue | <0.0001 |
| **Unweighted UniFrac** | Sediment | 0.34 | Sediment *vs* Gut content | <0.01 |
|  | Gut content | 0.38 | Gut content *vs* Gut tissue | <0.0001 |
|  | Gut tissue | 0.46 | Sediment *vs* Gut tissue | <0.0001 |

**Table S4: Results of Pairwise permanova comparisons among sites on bacterial community dissimilarity based on Bray-Curtis dissimilarity matrix for each habitat separately.**

| **Habitat** | **Site comparisons** | **F-statistics** | **R^2^** | **P-value** | **P-value adjusted** |
| --- | --- | --- | --- | --- | --- |
| **Sediment** | **South Shetland I. *vs* Kerguelen I.** | 78.33 | 0.90 | 0.004 | <0.01 |
|  | **South Shetland I. *vs* Patagonia** | 73.96 | 0.88 | 0.001 | <0.01 |
|  | **South Shetland I. *vs* South Georgia** | 28.39 | 0.74 | 0.003 | <0.01 |
|  | **South Shetland I. *vs* Falkland/Malvinas I.** | 68.92 | 0.88 | 0.003 | <0.01 |
|  | **Kerguelen I. *vs* Patagonia** | 34.67 | 0.79 | 0.002 | <0.01 |
|  | **Kerguelen I. *vs* South Georgia** | 18.30 | 0.67 | 0.001 | <0.01 |
|  | **Kerguelen I. *vs* Falkland/Malvinas I.** | 40.38 | 0.83 | 0.006 | <0.01 |
|  | **Patagonia *vs* Falkland/Malvinas I.** | 48.66 | 0.84 | 0.002 | <0.01 |
|  | **Patagonia *vs* South Georgia** | 16.60 | 0.62 | 0.004 | <0.01 |
|  | **South Georgia *vs* Falkland/Malvinas I.** | 21.18 | 0.70 | 0.004 | <0.01 |
| **Gut content** | **South Shetland I. *vs* Kerguelen I.** | 75.55 | 0.61 | 0.001 | <0.01 |
|  | **South Shetland I. *vs* Patagonia** | 86.61 | 0.55 | 0.001 | <0.01 |
|  | **South Shetland I. *vs* South Georgia** | 42.60 | 0.51 | 0.001 | <0.01 |
|  | **South Shetland I. *vs* Falkland/Malvinas I.** | 83.71 | 0.63 | 0.001 | <0.01 |
|  | **Kerguelen I. *vs* Patagonia** | 31.71 | 0.39 | 0.001 | <0.01 |
|  | **Kerguelen I. *vs* South Georgia** | 35.32 | 0.64 | 0.001 | <0.01 |
|  | **Kerguelen I. *vs* Falkland/Malvinas I.** | 77.02 | 0.73 | 0.001 | <0.01 |
|  | **Patagonia *vs* Falkland/Malvinas I.** | 42.39 | 0.46 | 0.001 | <0.01 |
|  | **Patagonia *vs* South Georgia** | 13.69 | 0.25 | 0.001 | <0.01 |
|  | **South Georgia *vs* Falkland/Malvinas I.** | 36.17 | 0.64 | 0.001 | <0.01 |
| **Gut tissue** | **South Shetland I. *vs* Kerguelen I.** | 4.52 | 0.18 | 0.001 | <0.01 |
|  | **South Shetland I. *vs* Patagonia** | 6.87 | 0.19 | 0.001 | <0.01 |
|  | **South Shetland I. *vs* South Georgia** | 5.91 | 0.20 | 0.001 | <0.01 |
|  | **South Shetland I. *vs* Falkland/Malvinas I.** | 6.48 | 0.19 | 0.001 | <0.01 |
|  | **Kerguelen I. *vs* Patagonia** | 4.23 | 0.18 | 0.001 | <0.01 |
|  | **Kerguelen I. *vs* South Georgia** | 4.96 | 0.28 | 0.001 | <0.01 |
|  | **Kerguelen I. *vs* Falkland/Malvinas I.** | 4.76 | 0.23 | 0.001 | <0.01 |
|  | **Patagonia *vs* Falkland/Malvinas I.** | 8.53 | 0.25 | 0.001 | <0.01 |
|  | **Patagonia *vs* South Georgia** | 5.66 | 0.20 | 0.001 | <0.01 |
|  | **South Georgia *vs* Falkland/Malvinas I.** | 6.82 | 0.26 | 0.001 | <0.01 |

**Table S5: Permutation test on the results of the variation partitioning analysis.** Permutation test was conducted using *anova.cca* function (*vegan* package) with 999 permutations. Geography is represented by PCNM1 and PCNM2 variables, and environment by PC1 and PC2 variables.

|  | Geography | | Environment | | |
| --- | --- | --- | --- | --- | --- |
|  | **F** | **p-value** | | **F** | **p-value** |
| Sediment | 45.2 | 0.001*** | | 36.4 | 0.001*** |
| Gut content | 28.4 | 0.001*** | | 22.7 | 0.001*** |
| Gut tissue | 3.2 | 0.001*** | | 2.9 | 0.001*** |


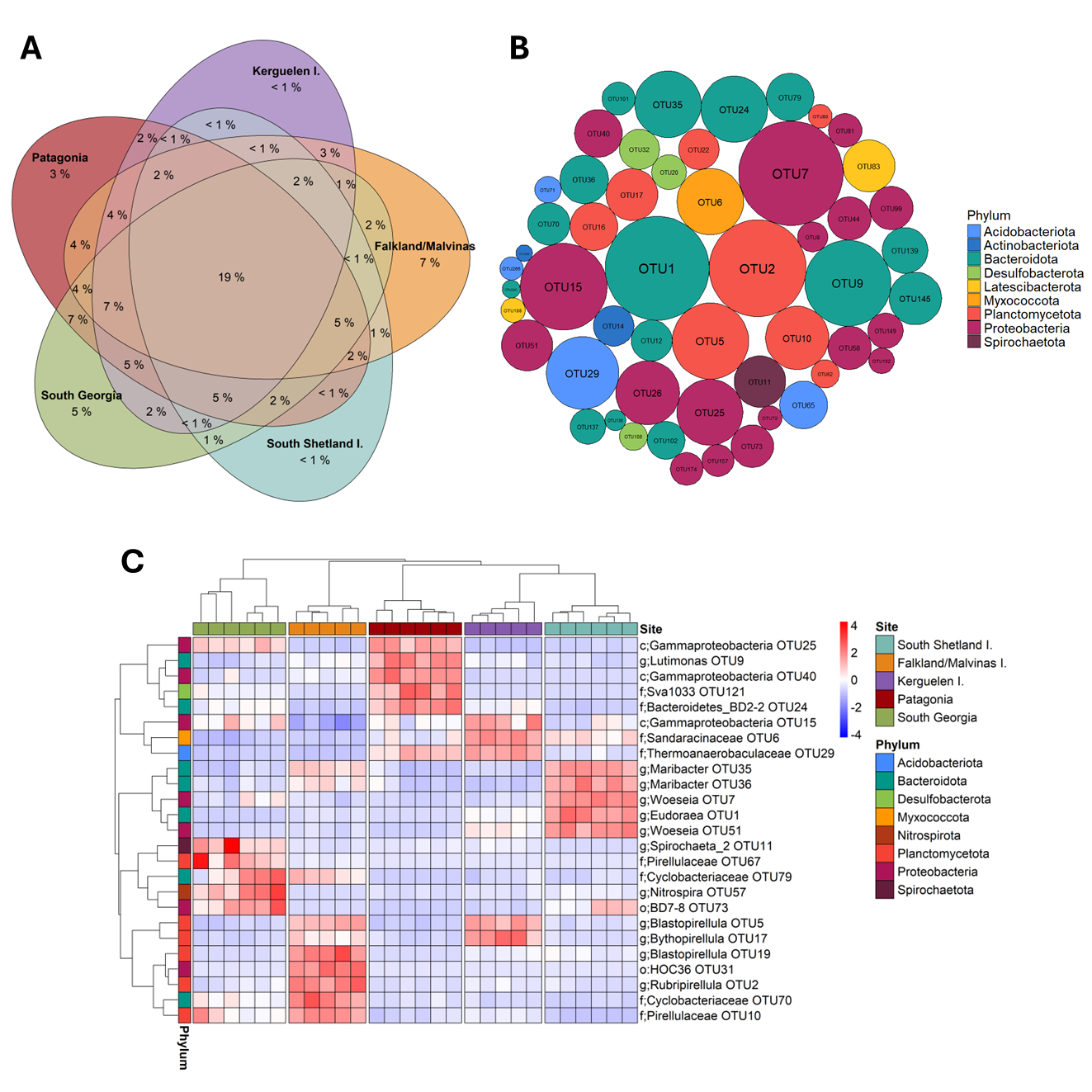


**Figure S4: Taxonomic differentiation of sediment microbiota**. A) Venn diagram of the percentage of OTUs shared among sites for the sediment bacterial communities. B) Circle packing visualization of sediment site’s core OTUs. Colours represent phyla, and size is proportional to their abundance in sediment samples. C) Heatmap visualizing the z-score distribution of the top five most discriminant OTUs for each site for the sediment bacterial communities. The dendrogram at the top demonstrates the hierarchical clustering of samples by site, and the dendrogram at the left shows the hierarchical clustering of the taxa. OTUs abundances were centred and scaled (row Z-score). These OTUs were identified using LEFse analysis, with only those taxa having a p-value < 0.01 (Wilcoxon rank-sum test) and LDA score > 2 being displayed. OTUs are indicated with their best taxonomic affiliation. Letters before the taxon name indicate the taxonomic level: p: Phylum, c: Class, o: Order, f: Family, g: Genus.


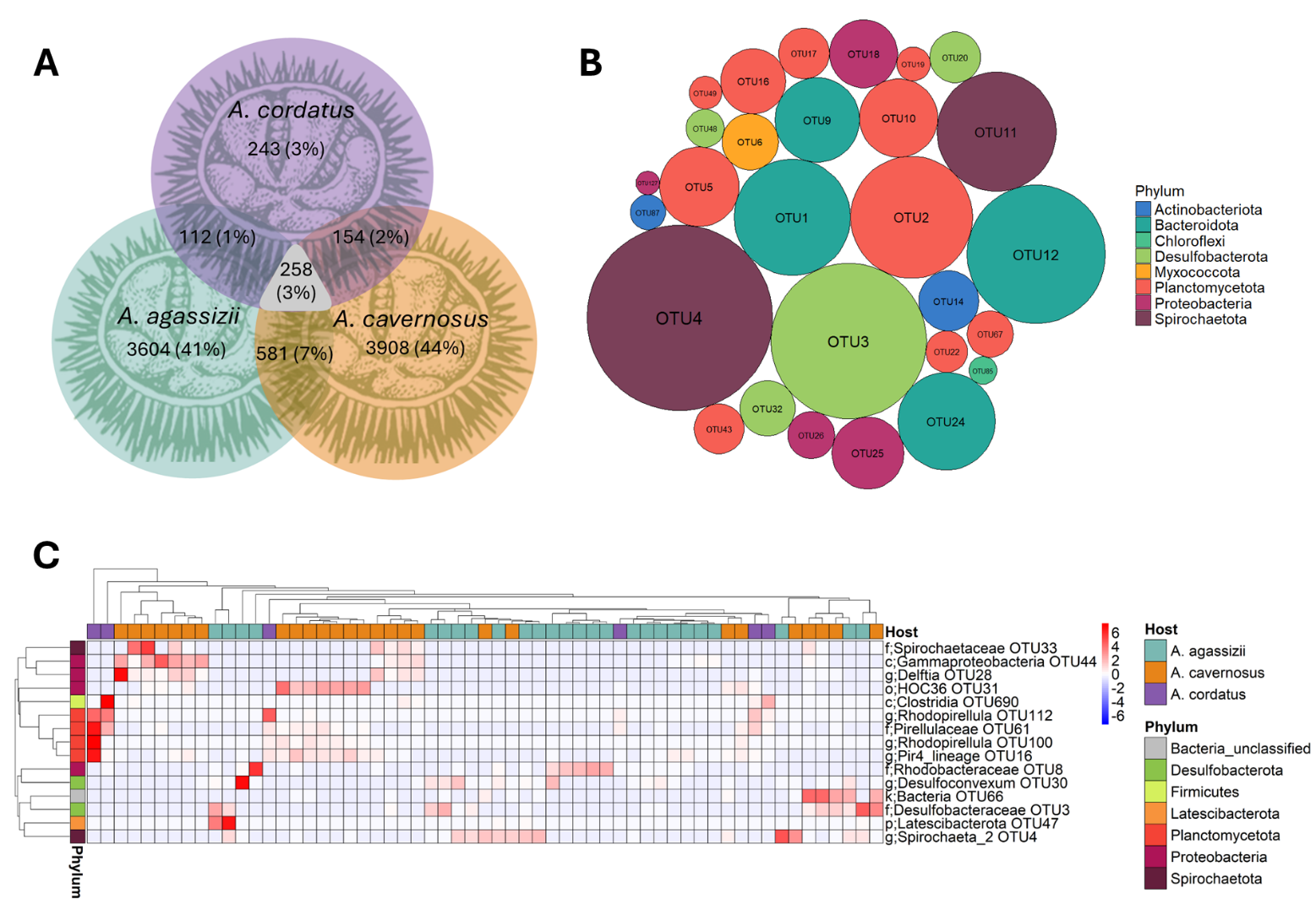


**Figure S5: Taxonomic differentiation of *Abatus* spp gut tissue microbiota.** A) Venn diagram displaying shared and specific OTUs within the gut tissues bacterial communities of the three *Abatus* species (based on the complete gut tissue OTU dataset). B) Circle packing visualization representing the *Abatus* core OTUs. Colours correspond to phylum and size of each circle is proportional to their abundance in gut tissue samples. C) Heatmap illustrating the z-score distribution of the top five most discriminant OTUs for each *Abatus* species in the gut tissue communities. OTUs abundances were centred and scaled (row Z-score).These OTUs were identified using LEFse analysis, with only those taxa having a p-value < 0.01 (Wilcoxon rank-sum test) and LDA score > 2 being displayed. OTUs are annotated with their best taxonomic affiliation. Letters before the taxon name indicate the taxonomic level: k (Kingdom), p (Phylum), c (Class), o (Order), f (Family) and g (Genus).

**Table S6: Ten most discriminant OTUs for each habitat.** OTUs were identified by a LEFse analysis (p-values < 0.01 (Wilcoxon rank-sum test) and LDA score effect size > 2) with their taxonomic annotation.

| Habitat | OTU | Phylum | Class | Order | Family | Genus | LDA score |
| --- | --- | --- | --- | --- | --- | --- | --- |
| Sediment | OTU7 | Proteobacteria | Gammaproteobacteria | Steroidobacterales | Woeseiaceae | *Woeseia* | 4.31 |
|  | OTU15 | Proteobacteria | Gammaproteobacteria | Gammaproteobacteria_Incertae_Sedis | Unknown_Family | *uncultured* | 4.06 |
|  | OTU29 | Acidobacteriota | Thermoanaerobaculia | Thermoanaerobaculales | Thermoanaerobaculaceae | *Subgroup_23* | 3.99 |
|  | OTU26 | Proteobacteria | Gammaproteobacteria | B2M28 | B2M28_fa | *B2M28_ge* | 3.77 |
|  | OTU83 | Latescibacterota | Latescibacterota_cl | Latescibacterota_or | Latescibacterota_fa | *Latescibacterota_ge* | 3.72 |
|  | OTU51 | Proteobacteria | Gammaproteobacteria | Steroidobacterales | Woeseiaceae | *Woeseia* | 3.71 |
|  | OTU79 | Bacteroidota | Bacteroidia | Cytophagales | Cyclobacteriaceae | *uncultured* | 3.67 |
|  | OTU65 | Acidobacteriota | Thermoanaerobaculia | Thermoanaerobaculales | Thermoanaerobaculaceae | *Subgroup_23* | 3.61 |
|  | OTU25 | Proteobacteria | Gammaproteobacteria | Gammaproteobacteria_Incertae_Sedis | Unknown_Family | *uncultured* | 3.60 |
|  | OTU73 | Proteobacteria | Gammaproteobacteria | BD7-8 | BD7-8_fa | *BD7-8_ge* | 3.57 |
| Gut content | OTU2 | Planctomycetota | Planctomycetes | Pirellulales | Pirellulaceae | *Rubripirellula* | 4.47 |
|  | OTU8 | Proteobacteria | Alphaproteobacteria | Rhodobacterales | Rhodobacteraceae | *Rhodobacteraceae_unclassified* | 4.20 |
|  | OTU9 | Bacteroidota | Bacteroidia | Flavobacteriales | Flavobacteriaceae | *Lutimonas* | 4.16 |
|  | OTU5 | Planctomycetota | Planctomycetes | Pirellulales | Pirellulaceae | *Blastopirellula* | 4.11 |
|  | OTU38 | Acidobacteriota | Vicinamibacteria | Subgroup_17 | Subgroup_17_fa | *Subgroup_17_ge* | 3.79 |
|  | OTU19 | Planctomycetota | Planctomycetes | Pirellulales | Pirellulaceae | *Blastopirellula* | 3.67 |
|  | OTU54 | Modulibacteria | Moduliflexia | Moduliflexales | Moduliflexaceae | *Moduliflexaceae_ge* | 3.64 |
|  | OTU22 | Planctomycetota | Planctomycetes | Pirellulales | Pirellulaceae | *Blastopirellula* | 3.62 |
|  | OTU44 | Proteobacteria | Gammaproteobacteria | Gammaproteobacteria_Incertae_Sedis | Unknown_Family | *uncultured* | 3.59 |
|  | OTU32 | Desulfobacterota | Desulfobulbia | Desulfobulbales | Desulfocapsaceae | *SEEP-SRB4* | 3.51 |
| Gut tissue | OTU12 | Bacteroidota | Bacteroidia | Flavobacteriales | Flavobacteriaceae | *Lutibacter* | 4.40 |
|  | OTU28 | Proteobacteria | Gammaproteobacteria | Burkholderiales | Comamonadaceae | *Delftia* | 4.31 |
|  | OTU33 | Spirochaetota | Spirochaetia | Spirochaetales | Spirochaetaceae | *Spirochaetaceae_unclassified* | 4.02 |
|  | OTU95 | Bacteria_unclassified | Bacteria_unclassified | Bacteria_unclassified | Bacteria_unclassified | *Bacteria_unclassified* | 3.70 |
|  | OTU47 | Latescibacterota | Latescibacterota_cl | Latescibacterota_or | Latescibacterota_fa | *Latescibacterota_ge* | 3.70 |
|  | OTU55 | Firmicutes | Clostridia | Clostridia_unclassified | Clostridia_unclassified | *Clostridia_unclassified* | 3.59 |
|  | OTU203 | Bacteroidota | Bacteroidia | Flavobacteriales | Flavobacteriaceae | *Lutibacter* | 3.53 |
|  | OTU74 | Desulfobacterota | Desulfarculia | Desulfarculales | Desulfarculaceae | *Dethiosulfatarculus* | 3.51 |
|  | OTU66 | Bacteria_unclassified | Bacteria_unclassified | Bacteria_unclassified | Bacteria_unclassified | *Bacteria_unclassified* | 3.44 |
|  | OTU41 | Spirochaetota | Spirochaetia | Spirochaetales | Spirochaetaceae | *Spirochaeta_2* | 3.42 |

**Table S7: Ecological processes driving the assembly and shift of bacterial communities in each habitat within and among sites.** Values represent the contribution of each process to the bacterial assembly for each habitat and within and among sites.

|  |  | Stochastics processes | | | | Deterministic processes | | | |
| --- | --- | --- | --- | --- | --- | --- | --- | --- | --- |
|  |  | Homogenizing dispersal | Dispersal limitation | Ecological drift | **Total explained by stochastic processes** | | Homogeneous selection | Variable selection | **Total explained by deterministic processes** |
| Intra-site | **Sediment** | 76.7% | 0.0% | 9.3% | **86.0%** | | 0.0% | 14.0% | **14.0%** |
|  | **Gut content** | 36.0% | 17.2% | 22.6% | **75.8%** | | 8.5% | 15.6% | **24.1%** |
|  | **Gut tissue** | 5.4% | 18.8 % | 38.9% | **63.1%** | | 13.4% | 23.4% | **36.8%** |
| Inter-site | **Sediment** | 0.0% | 8.7% | 0.0% | **8.7%** | | 0.0% | 91.3% | **91.3%** |
|  | **Gut content** | 0.0% | 22.9% | 8.3% | **31.2%** | | 3.9% | 64.8% | **68.4%** |
|  | **Gut tissue** | 0.4% | 22.9% | 9.2% | **32.5%** | | 0.3% | 68.3% | **68.6%** |

**Table S8: Wilcoxon test between site means of the percentage of each assemblage process**. Ecological processes were estimated within each site (n=5) for each habitat. Then the mean values of each ecological process across sites were compared between habitats with Wilcoxon non-parametric test.

|  | **Intra-site comparison** | | | **Inter-site comparison** | | | | |
| --- | --- | --- | --- | --- | --- | --- | --- | --- |
|  | **Sediment *vs* Gut tissue** | **Sediment *vs* Gut content** | **Gut content *vs* Gut tissue** | | **Sediment *vs* Gut tissue** | **Sediment *vs* Gut content** | **Gut content *vs* Gut tissue** |  |
| **Dispersal limitation** | 0.025 * | 0.070 ns | 0.390 ns | | 0.0182 ** | 0.113 ns | 0.623 ns |  |
| **Ecological drift** | 0.020 * | 0.240 ns | 0.055 ns | | 0.0007 *** | 0.006 ** | 0.301 ns |  |
| **Homogenizing dispersal** | 0.008 ** | 0.550 ns | 0.310 ns | | 0.168 ns | NA | 0.078 ns |  |
| **Variable selection** | 0.298 ns | 0.680 ns | 0.420 ns | | 0.004 ** | 0.082 ns | 0.734 ns |  |
| **Homogeneous selection** | 0.007 ** | 0.025 * | 0.540 ns | | 0.0778 ns | 0.0778 ns | 0.456 ns |  |


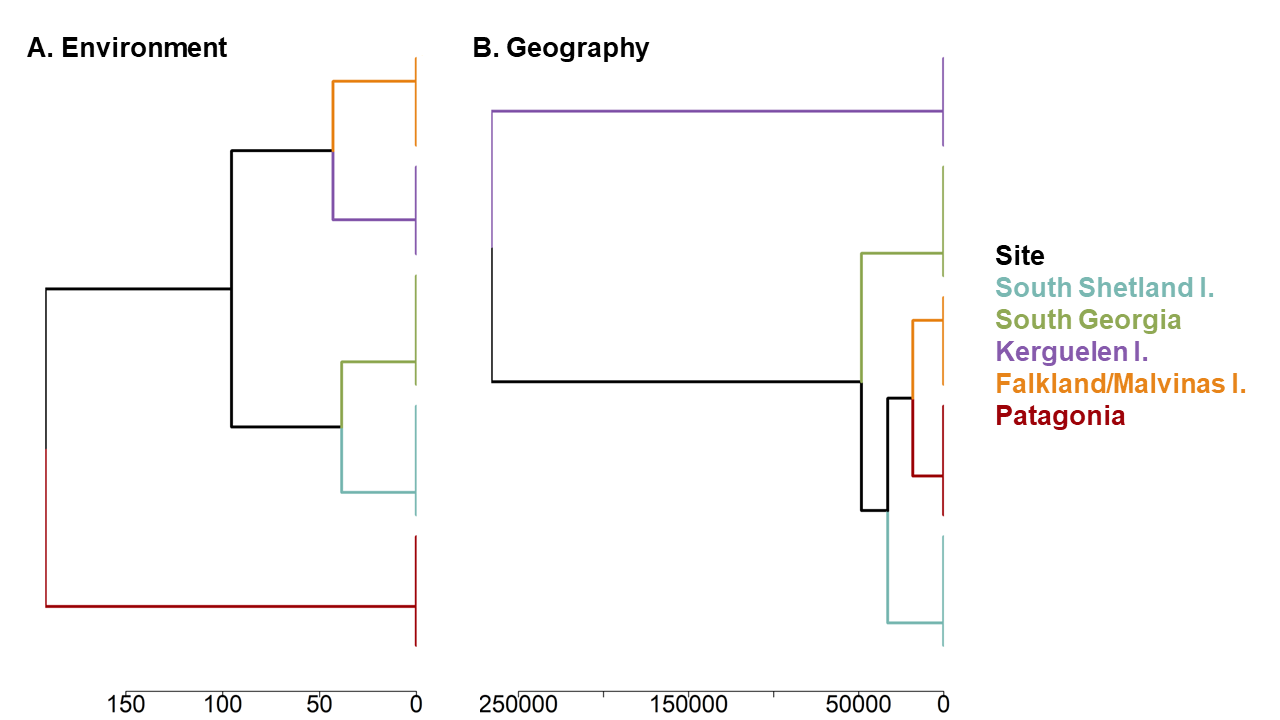


**Figure S6**: **Environmental and Geographical hierarchical clustering dendrogram analysis of sediment samples**. Hierarchical clustering dendrogram analysis using Ward method and environmental and geographical distances for sediment samples. Colors represent the sample’s site.


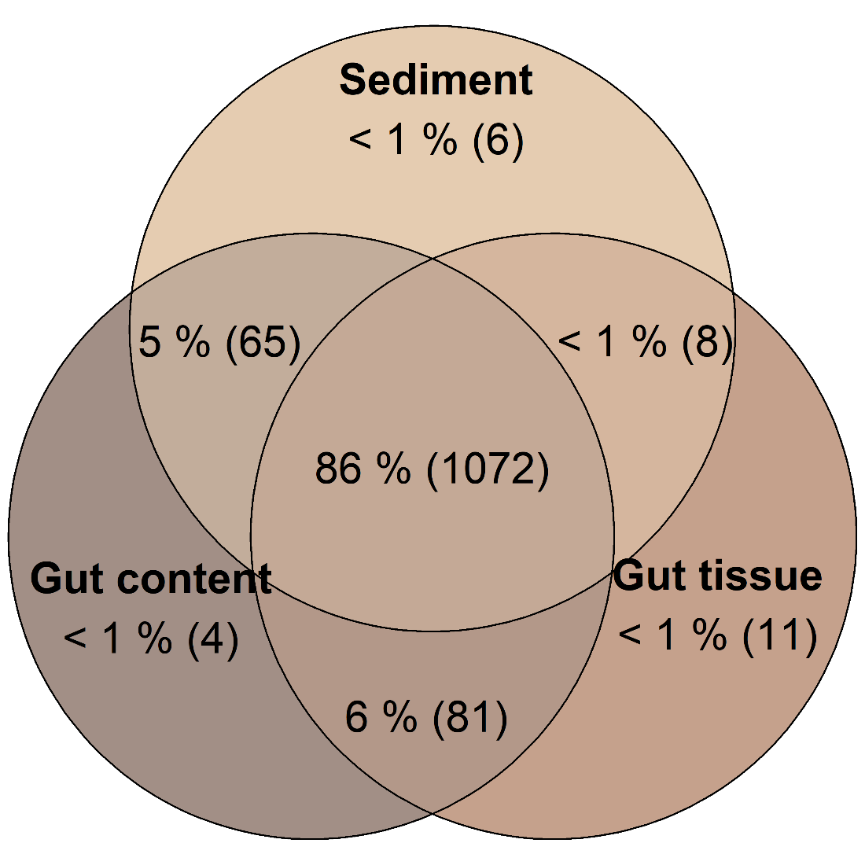


**Figure S7: Shared and specific OTUs among habitats.** Venn diagram displaying the proportion of shared and specific OTUs among the three habitats across all sites, in percentage relative to the total OTU number of the complete dataset. The numbers in parenthesis are the absolute numbers of shared and specific OTUs among the three habitats. The corresponding relative abundances of these shared and specific OTUs are provided in Supplementary Table S9.

**Table S9**: Number and abundance of OTUs shared between sediment and gut tissue habitat.

|  | Number of OTUs | Abundance (number of sequences) | Relative abundance (% the gut tissue sequence number) |
| --- | --- | --- | --- |
| OTUs shared between sediment and gut tissue | 1080 | 320 351 | 90.49% |
| OTUs only present in the gut tissue | 11 | 1473 | 0.42% |
| Total of OTUs present in gut tissue | 1172 | 354 000 | 100.00% |

**Supplementary Methods S1: DNA extraction and PCR amplification of eggs, gastrulas and juveniles supports the horizontal transmission hypothesis in *Abatus* sea urchins.**

DNA from eggs, gastrula, juveniles and gut tissue samples of *A. agassizii* from Fildes Bay, King George Island (South Shetland, Antarctic Peninsula) were extracted with the DNeasy® PowerSoil® Pro Kit (Qiagen, Hilden, Germany), the DNeasy® Blood & Tissue Kit and the traditional salt extraction protocol (Aljanabi 1997). The V4-V5 region of the 16S rRNA gene was amplified as described in the manuscript. The PCR products were checked by electrophoresis on an agarose gel (1%).

| Sample name | Sample type | DNA extraction method | [DNA] (ng/µl) | V3-V4 16S rRNA gene PCR amplification |
| --- | --- | --- | --- | --- |
| Gut tissue 1 | Gut tissue | DNeasy® PowerSoil® Pro Kit, Qiagen | Under detection limit | Yes |
| Gut tissue 2 | Gut tissue |  | 8.3 | Yes |
| Gut tissue 3 | Gut tissue |  | 3.9 | Yes |
| Gut tissue 4 | Gut tissue |  | 194.0 | Yes |
| Gut tissue 5 | Gut tissue |  | 67.5 | Yes |
| Eggs 1 | Eggs |  | 1.4 | No |
| Gastrula 1 | Gastrula |  | Under detection limit | No |
| Eggs 2 | Eggs | DNeasy® Blood & Tissue Kit, Qiagen | 1.4 | No |
| Gastrula 2 | Gastrula |  | Under detection limit | No |
| Juveniles 2 | Juveniles |  | 1.4 | No |
| Juveniles | Juveniles | Salt extraction protocol (Aljanabi & Martinez 1997) | 55.8 | Yes |
| Eggs 8 | Eggs |  | 7.1 | No |
| Gastrula 8 | Gastrula |  | 38.5 | No |

**Table S10:** Summary of DNA extraction method, DNA concentrations (ng/µl) and PCR amplification result for gut tissue, juveniles, eggs and gastrula samples of *Abatus* sea urchins. DNA quantification by Qubit® 3.0. Fluorometer (ThermoFisher Scientific, Lithuania). Different DNA extraction protocols were tested. Eggs referred to the whole gonads inside of female *Abatus* and gastrula referred to single eggs found in the marsupial pouches (Schatt and Feral 1996).

DNA extraction results showed that very small amounts of DNA were extracted from eggs, and gastrulas compared to juveniles and gut tissue from adults sea urchins, independently of the used DNA extraction method (Table S1). The PCR amplification of prokaryotic 16S rRNA gene failed for eggs and gastrulas samples, while it was successful for gut tissue samples of juveniles and adults (Figure S1). These results suggest that even if there is a bacterial community transmitted through the eggs, it is minor and undetectable through our PCR protocol. Thus these results reinforce the hypothesis that the vertical bacterial transmission from mother to offspring in *Abatus* sea urchin is minimal and strongly suggests that *Abatus* sea urchins acquire their gut microbiota mainly from the environment (horizontal transmission) once the digestive tube opens at the juvenile stage (Schatt and Feral 1996).


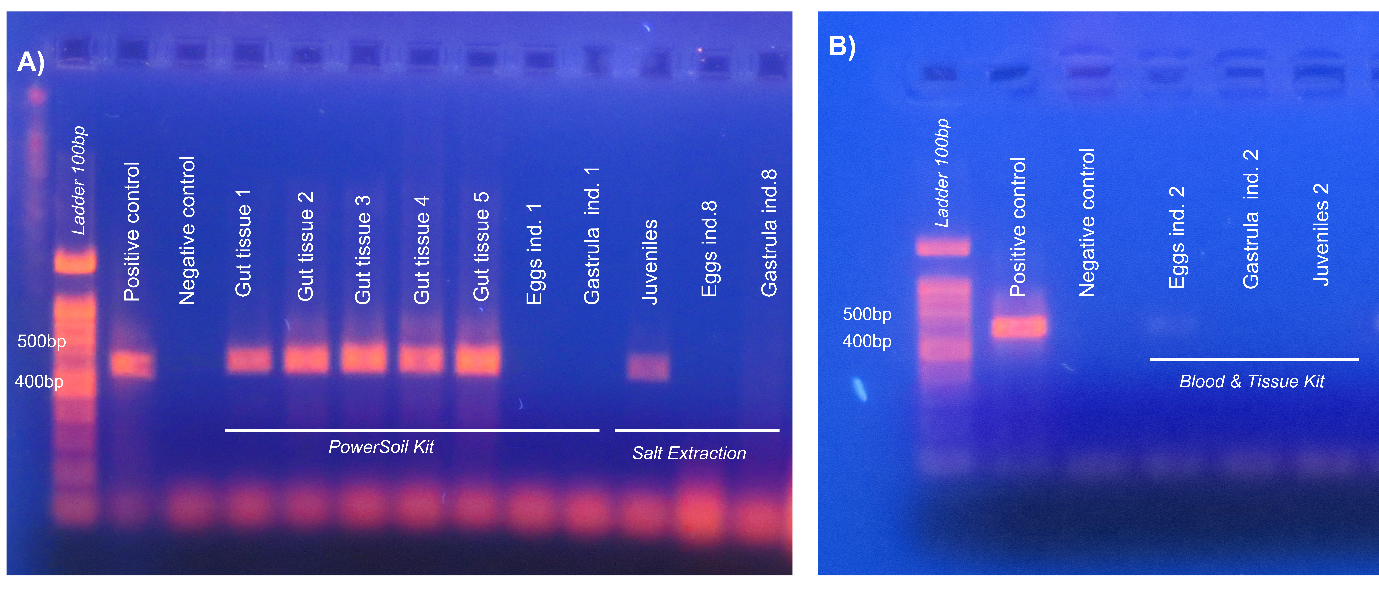
**Figure S8:** Picture of an electrophoresis gel showing the results of the V4-V5 16s rRNA gene PCR amplification for gut tissue, eggs, gastrula and juveniles samples extracted with A) DNeasy® PowerSoil kit and Salt extraction methods and B) DNeasy® Blood & Tissue Kit, Qiagen. The numbers in the sample names refer to the identification of each *Abatus* individual.

**Supplementary Material S1:** Biogeographical distribution of sediment-specific OTUs

Most bacterial taxa discriminating our sediment communities have been previously reported in marine benthic ecosystems (Kumar *et al.* 2012; Dyksma *et al.* 2016a; Mußmann *et al.* 2017; Hoffmann *et al.* 2020), while others differ from the bacterial composition previously reported in sampling sites close to ours (e.g. in King George Island and South Georgia (Wang *et al.* 2017; Wunder *et al.* 2021)), which could be attributed to the bioturbation activity of the dense sea urchin populations in our sampling localities (Lohrer *et al.* 2005; Thompson and Riddle 2005). The major sediment-specific OTUs belong to *Woeseia* (Proteobacteria), *Cyclobacteriaceae* (Bacteroidota)*, Thermoanaerobaculaceae* (Acidobacteriota)*,* Gammaproteobacteria BD7-8 and Latescibacterota. Some of these taxa have been consistently reported in marine environments similar to our sampling locations and might be involved in important roles such as remineralization and cycling of nutrients. For instance, *Woeseia* has been described in marine sediment (Du *et al.* 2016; Mußmann *et al.* 2017; Hinger, Pelikan and Mußmann 2019; Hoffmann *et al.* 2020), and comprised halophilic facultative anaerobes (Du *et al.* 2016). *Cyclobacteriaceae* members are nitrate reducers (Kumar *et al.* 2012; Srinivas *et al.* 2014). Members of the Gammaproteobacteria BD7-8 order are chemoautotrophic aerobic sulphide oxidizers (Kleiner *et al.* 2012), ubiquitous in benthic habitats (Dyksma *et al.* 2016b), and one member (γ3) has been described as a symbiont of *Olavius algarvensis* oligochaete (Woyke *et al.* 2006). South Georgia surface sediment (distinct from our sampling sites) were characterized by the presence of Flavobacteriales, *Rhodobacteraceae*, *Haliaceae*, *Pirellulaceae* and *Rubritaleaceae*, but their relative abundance decreased with sediment depth (Wunder *et al.* 2021). Wang *et al.* studied the intertidal sediment from Fildes Peninsula in King George Island and found that members of Proteobacteria (such as *Psychrobacter*, *Psychromonas* and *Granulosicoccus*) and Bacteroidetes (such as *Polaribacter* and *Maribacter*) phyla were the most dominant, representing over 90% of the total community (Wang *et al.* 2017). However, they did not find *Woeseia* genus in their samples while in our study this genus was not only present but discriminant of the sediments from Fildes Bay, King George Island, South Shetland Islands. The benthic ecosystems studied here are different from the ones studied in Wang *et al.* (Wang *et al.* 2017), and comprised sediment patches that are densely populated by *Abatus* sea urchins that, thanks to their bioturbation activity (Lohrer *et al.* 2005; Thompson and Riddle 2005), can modify the local environment and the bacterial communities, explaining why we found different bacterial taxa.

**Supplementary Material S2: Biogeographical classification of South Georgia biota**

Historically, all islands south of the APF, such as South Georgia, have been considered to belong to the Antarctic province from an oceanographic point of view (Deacon 1982; Orsi, Whitworth and Nowlin 1995). However, when analysing the distribution of marine macroorganisms, the biogeographical classification of South Georgia biota remains a topic of discussion. The distribution of some benthic taxonomic groups (Gastropoda, Bivalvia, Cheilostomata, Pycnogonida and Ascidiacea) supports the notion that South Georgia is affiliated with the Antarctic province (Linse *et al.* 2006; Primo and Vázquez 2007; Griffiths, Barnes and Linse 2009; Griffiths *et al.* 2011). Conversely, for other taxa (Porifera, Cyclostomata, Echinoidea), South Georgia shares more similarity with Subantarctic regions, highlighting this site as a transition zone between the Magellanic and the Antarctic regions (e.g .Downey *et al.* 2012; Broyer and Koubbi 2014; Griffiths and Waller 2016). Here, taking into account the biogeographic distribution of *Abatus*, South Georgia should be considered as part of the Maritime Antarctic province since both sites host *A. agassizii* (David *et al.* 2005). Moreover, our sampling sites from South Georgia and South Shetland display the highest similarity in terms of environmental parameters of the superficial coastal seawater (Figure S6).

**Additional References**

Aljanabi S. Universal and rapid salt-extraction of high quality genomic DNA for PCR- based techniques. *Nucleic Acids Research* 1997;**25**:4692–3.

Broyer C de, Koubbi P. Conclusions: Present and Future of Southern Ocean Biogeography. *Biogeographic Atlas of the Southern Ocean*. Cambridge: Published by The Scientific Committee on Antarctic Research, Scott Polar reseach Institute, 2014.

David B, Choné T, Mooi R *et al.* Antarctic echinoidea. Synopses of the Antarctic benthos. *Lichtenstein: ARG Gantner* 2005.

Deacon GER. Physical and biological zonation in the Southern Ocean. *Deep Sea Res Part I Oceanogr Res Pap* 1982;**29**:1–15.

Downey RV, Griffiths HJ, Linse K *et al.* Diversity and Distribution Patterns in High Southern Latitude Sponges. Thrush S (ed.). *PLoS ONE* 2012;**7**:e41672.

Du Z-J, Wang Z-J, Zhao J-X *et al.* Woeseia oceani gen. nov., sp. nov., a chemoheterotrophic member of the order Chromatiales, and proposal of Woeseiaceae fam. nov. *International Journal of Systematic and Evolutionary Microbiology* 2016;**66**:107–12.

Dyksma S, Bischof K, Fuchs BM *et al.* Ubiquitous Gammaproteobacteria dominate dark carbon fixation in coastal sediments. *ISME J* 2016a;**10**:1939–53.

Dyksma S, Bischof K, Fuchs BM *et al.* Ubiquitous Gammaproteobacteria dominate dark carbon fixation in coastal sediments. *ISME J* 2016b;**10**:1939–53.

Griffiths HJ, Arango CP, Munilla T *et al.* Biodiversity and biogeography of Southern Ocean pycnogonids. *Ecography* 2011;**34**:616–27.

Griffiths HJ, Barnes DKA, Linse K. Towards a generalized biogeography of the Southern Ocean benthos. *J Biogeogr* 2009;**36**:162–77.

Griffiths HJ, Waller CL. The first comprehensive description of the biodiversity and biogeography of Antarctic and Sub-Antarctic intertidal communities. *Journal of Biogeography* 2016;**43**:1143–55.

Hoffmann K, Bienhold C, Buttigieg PL *et al.* Diversity and metabolism of Woeseiales bacteria, global members of marine sediment communities. *ISME J* 2020;**14**:1042–56.

Kleiner M, Wentrup C, Lott C *et al.* Metaproteomics of a gutless marine worm and its symbiotic microbial community reveal unusual pathways for carbon and energy use. *Proc Natl Acad Sci USA* 2012;**109**, DOI: 10.1073/pnas.1121198109.

Kumar AP, Aravind R, Francis K *et al.* Shivajiella indica gen. nov., sp. nov., a marine bacterium of the family “Cyclobacteriaceae” with nitrate reducing activity. *Systematic and Applied Microbiology* 2012;**35**:320–5.

Linse K, Griffiths HJ, Barnes DKA *et al.* Biodiversity and biogeography of Antarctic and sub-Antarctic mollusca. *Deep Sea Res Part II Top Stud Oceanogr* 2006;**53**:985–1008.

Lohrer AM, Thrush SF, Hunt L *et al.* Rapid reworking of subtidal sediments by burrowing spatangoid urchins. *J Exp Mar Biol Ecol* 2005;**321**:155–69.

Mußmann M, Pjevac P, Krüger K *et al.* Genomic repertoire of the Woeseiaceae/JTB255, cosmopolitan and abundant core members of microbial communities in marine sediments. *ISME J* 2017;**11**:1276–81.

Orsi AH, Whitworth T, Nowlin WD. On the meridional extent and fronts of the Antarctic Circumpolar Current. *Deep Sea Research Part I: Oceanographic Research Papers* 1995;**42**:641–73.

Primo C, Vázquez E. Zoogeography of the Antarctic ascidian fauna in relation to the sub-Antarctic and South America. *Antarct Sci* 2007;**19**:321–36.

Schatt P, Feral J-P. Completely Direct Development of Abatus cordatus, a Brooding Schizasterid (Echinodermata: Echinoidea) from Kerguelen, with Description of Perigastrulation, a Hypothetical New Mode of Gastrulation. *Biological Bulletin* 1996;**190**:24–44.

Srinivas TNR, Aditya S, Bhumika V *et al.* Lunatimonas lonarensis gen. nov., sp. nov., a haloalkaline bacterium of the family Cyclobacteriaceae with nitrate reducing activity. *Systematic and Applied Microbiology* 2014;**37**:10–6.

Thompson B, Riddle M. Bioturbation behaviour of the spatangoid urchin Abatus ingens in Antarctic marine sediments. *Mar Ecol Prog Ser* 2005;**290**:135–43.

Wang L, Liu X, Yu S *et al.* Bacterial community structure in intertidal sediments of Fildes Peninsula, maritime Antarctica. *Polar Biol* 2017;**40**:339–49.

Woyke T, Teeling H, Ivanova NN *et al.* Symbiosis insights through metagenomic analysis of a microbial consortium. *Nature* 2006;**443**:950–5.

Wunder LC, Aromokeye DA, Yin X *et al.* Iron and sulfate reduction structure microbial communities in (sub-)Antarctic sediments. *ISME J* 2021;**15**:3587–604.
